# Mortise‐Tenon Joint Inspired Weakly Solvated Gel Electrolyte Based on Halogen Bonds for High‐Voltage Lithium Metal Batteries

**DOI:** 10.1002/advs.202518448

**Published:** 2025-11-16

**Authors:** Shuohan Liu, Wensheng Tian, Hui Pan, Shunwei Chen, Xiujun Han, Hengdao Quan, Shenmin Zhu

**Affiliations:** ^1^ State Key Laboratory of Metal Matrix Composites School of Materials Science and Engineering Shanghai Jiao Tong University Shanghai 200240 China; ^2^ State Key Laboratory of Space Power‐Sources Shanghai Institute of Space Power‐Sources Shanghai 200245 China; ^3^ School of Materials Science and Engineering Qilu University of Technology (Shandong Academy of Sciences) Jinan Shandong 250353 China; ^4^ School of Materials and Chemistry University of Shanghai for Science and Technology Shanghai 200093 China

**Keywords:** functional polymer frameworks, gel electrolytes, lithium metal batteries, weak solvation structures

## Abstract

In situ gel polymer electrolytes (GPEs) offer a promising approach to improve safety and cycling stability in lithium metal batteries (LMBs), yet often suffer from poor electrode compatibility, especially at high temperatures. This work reports a “mortise‐tenon joint” inspired non‐covalent interaction — “π‐hole” based halogen bond, that enables solvation structure regulation beyond traditional van der Waals force or hydrogen bond. The electrophilic π‐hole on the polymer skeleton engages in halogen bonding with solvents, thereby weakening their coordination with Li^+^ to form a weakly solvated structure. Moreover, the fluorine‐rich skeleton participates in the formation of the electrode‐electrolyte interphases, achieving good anode compatibility and high‐voltage stability simultaneously. The resulting electrolyte exhibits high ionic conductivity (0.30 mS cm^−1^) and a Li^+^ transference number of 0.84. Li symmetric cells stably cycle over 1000 h. The Li/LiNi_0.8_Co_0.1_Mn_0.1_O_2_ (NCM811) cell delivers discharge specific capacities of 161.0 mAh g^−1^ after 500 cycles at 1C. Especially, the cell can work stably for 100 cycles at 80 °C (1C, 156.0 mAh g^−1^). Furthermore, the pouch cell achieves an energy density of 462.2 Wh kg^−1^. This study demonstrates that the concept of a weakly solvated gel electrolyte based on halogen bonds provides a new approach to achieving high‐energy‐density LMBs.

## Graphical abstract

Inspired by “mortise‐tenon joints”, a halogen bond‐mediated solvation regulation strategy is proposed for gel polymer electrolytes. This approach utilizes π‐hole framework directed halogen bond interactions to weaken solvent‐Li^+^ coordination, thereby inducing an anion‐dominated weakly solvating structure that facilitates the formation of a highly ion‐conductive and stable interfacial phase.

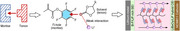

## 1 Introduction

Lithium metal batteries (LMBs) hold great promise for next‐generation energy storage, owing to their ultrahigh theoretical energy density exceeding 450 Wh kg^−1^.^[^ ^1^ ^]^ However, safety concerns associated with conventional liquid electrolytes, e.g., propensity for leakage and flammability, pose great barriers to their commercialization.^[^ ^2^, ^3^ ^]^ Solid‐state electrolytes appear to resolve this problem effectively, yet their low ionic conductivity and poor interfacial contact remain great challenges.^[^ ^4^, ^5^ ^]^ Gel polymer electrolytes (GPEs), which combine the ionic conductivity of liquids and the safety of solids, have emerged as a prospective alternative.^[^ ^6^, ^7^ ^]^ Unfortunately, existing GPEs face two major limitations: 1) insufficient inhibition of Li dendrites and deleterious reactions at the anode side, and 2) poor compatibility with high‐nickel cathodes (e.g., LiNi_0.8_Co_0.1_Mn_0.1_O_2_, NCM811).^[^ ^8^ ^]^ Therefore, simultaneously addressing the challenges at both the anode and cathode sides in a GPE battery is indeed crucial for realizing practical high‐energy‐density LMBs.

**Figure 1:**
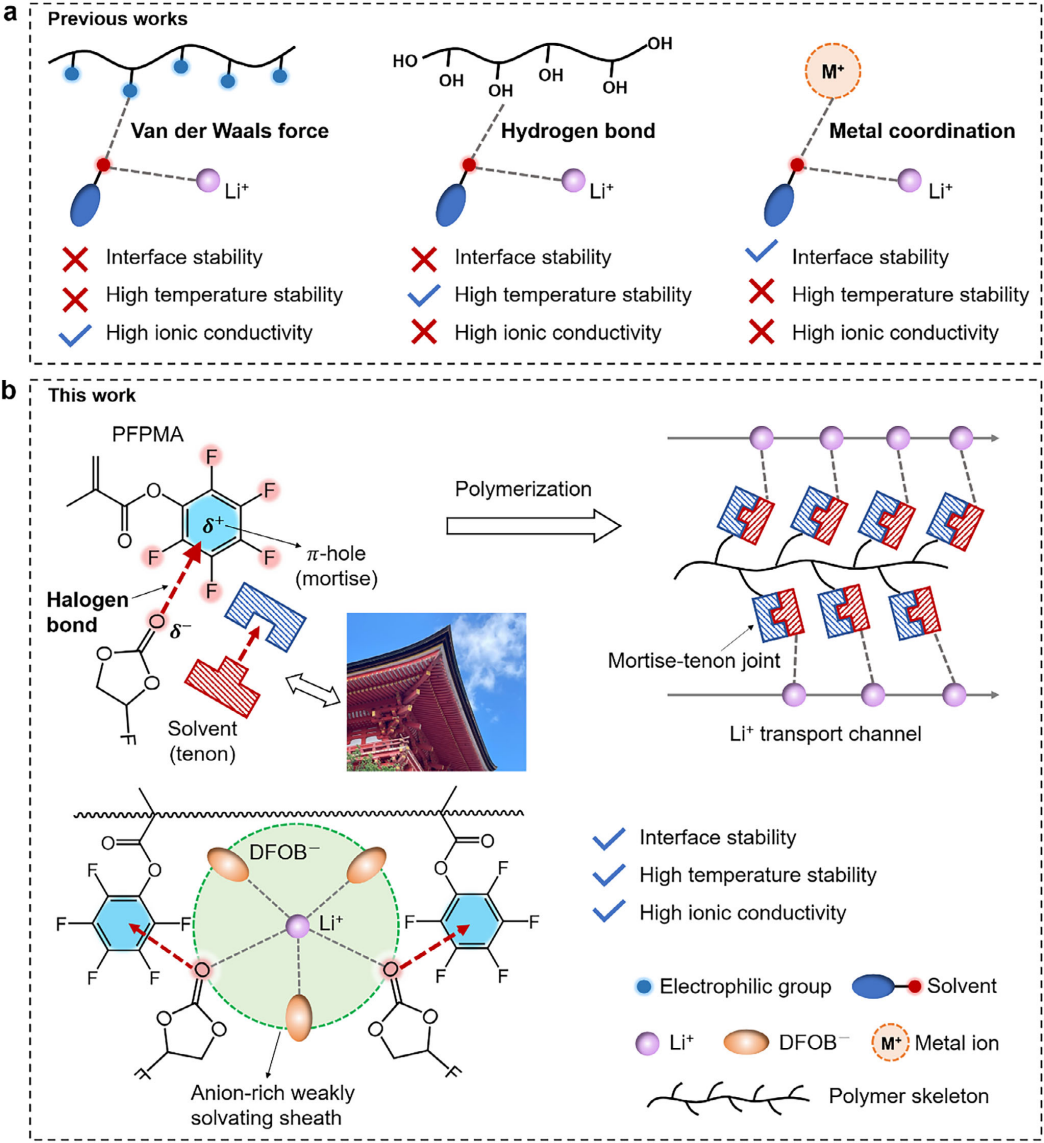
Conceptual design of “mortise‐tenon joint” inspired weakly solvated GPE. a) Previous work regulating solvation structure based on traditional interaction forces. b) Weak solvation structure induced by π‐hole‐based halogen bond interactions.

Reducing the affinity between Li^+^ and the solvent to create a weakly solvating environment promotes the formation of anion‐derived, inorganic‐rich electrode‐electrolyte interphases (EEIs), which is widely recognized as a key strategy to enhance GPE performance.^[^ ^7^, ^9^ ^]^ Recent studies have proposed various strategies to construct a weakly solvating environment in GPEs by introducing functional polymer frameworks or additives.^[^ ^10^, ^11^, ^12^, ^13^, ^14^, ^15^ ^]^ Generally, these approaches mainly depend on conventional non‐covalent interactions, e.g., van der Waals forces, hydrogen bonds, or metal coordination, which show limited utility in GPEs (**Figure** **1**a).^[^ ^16^ ^]^ The van der Waals forces can maintain high ionic conductivity of the electrolyte, but they are usually insufficient to effectively regulate the Li^+^ solvation structure for the relatively low binding energy (<10 kJ mol^−1^). Furthermore, due to their significant temperature‐dependent characteristic, van der Waals forces cannot ensure the high‐temperature stability for GPEs.^[^ ^17^ ^]^ Hydrogen bonds with stronger binding energy (10–40 kJ mol^−1^) can effectively enhance the high‐temperature stability of GPEs, but their unstable terminal hydroxyl groups may initiate undesirable side reactions, thereby compromising interfacial stability.^[^ ^18^, ^19^ ^]^ Metal coordination‐induced weakly solvating structures facilitate the formation of a stable interface, whereas strong interactions would lead to reduced ionic conductivity. In addition, such interactions require specific solvation conditions, as they are susceptible to disruption by strong electrostatic forces from the electrolyte.^[^ ^20^ ^]^ Thus, designing new strategies to surpass traditional non‐covalent interactions for regulating solvation structures in GPEs remains a significant challenge.

Halogen bonding is a novel type of non‐covalent interaction formed between halogen atoms and negatively charged Lewis bases, exhibiting strength comparable to hydrogen bonding.^[^ ^21^ ^]^ For instance, in halogenated benzene rings, the strong electron‐withdrawing effect of halogens generates a positive electrostatic potential at the ring center — termed the “π‐hole”.^[^ ^21^, ^22^ ^]^ When interacting with nucleophilic groups (e.g., C═O), the C═O bond engages with the π‐hole along the direction perpendicular to the π‐skeletal plane, resulting in a directional matching analogous to a “mortise‐tenon joint” structure. The interaction strength of this architecture is significantly stronger than conventional van der Waals forces. Critically, it lacks active hydrogens that participate in electrode reactions and is resistant to interference from electrostatic interactions of electrolyte ions. These properties render halogen bonding an ideal non‐covalent interaction for solvation structure regulation. Inspired by the mortise‐tenon joint, we design a halogen bonding interaction between the π‐holes on the polymer skeleton and solvent molecules to regulate Li^+^ solvation structure in GPEs (Figure 1b).

In this work, we report a π‐hole‐engineered GPE (FTPM) by using pentafluorophenyl methacrylate (PFPMA) as the polymer precursor. The strong electron‐withdrawing F atoms in PFPMA form the so‐called π‐holes at the center of the fluorobenzene rings. The π‐holes on the fluorinated polymer side chain exhibit dual functionality: 1) weakening Li⁺‐solvent coordination via halogen bonds (O→π‐hole), thus facilitating anion entry into the Li⁺ solvation sheath to form anion‐rich coordination structures; and 2) providing rapid ion transport channels and promoting the formation of inorganic‐rich EEIs. As is designed, the resulting electrolyte exhibits simultaneously high ionic conductivity and excellent interface stability. The assembled Li/NCM811 cell delivers a discharge capacity of 190.5 mAh g^−1^ at 4.5 V cutoff and retains 84.5% capacity after 500 cycles. Even under harsh conditions (80 °C, 1 C rate), the Li/NCM811 cells retain a capacity of 156.0 mAh g^−1^ after 100 cycles, with an ultra‐low decay rate of only 0.14% per cycle. This work pioneers the use of π‐hole electronic effects to endow the polymer framework with the function of solvation regulation, providing a novel paradigm for designing high‐performance electrolytes that simultaneously achieve high‐voltage endurance and high‐temperature stability.

## 2 Results and Discussion

### 2.1 Weak Solvation Structure Triggered by π‐Hole‐Based Halogen Bond Interactions

**Figure 2:**
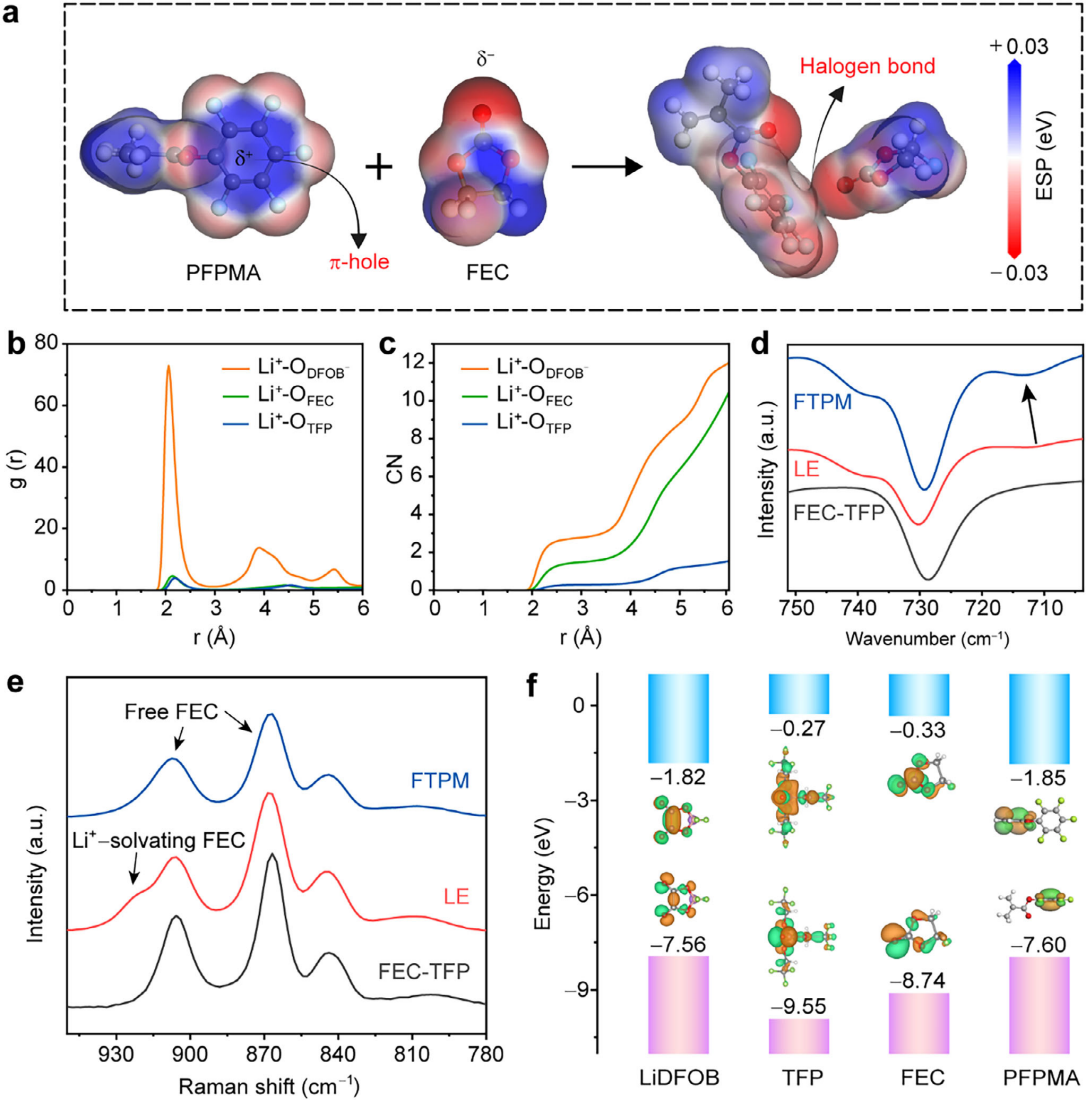
Solvation structure of FTPM. a) ESP of PFPMA, FEC, and PFPMA‐FEC (C: gray; F: pale blue; O: red; H: white). b,c) RDFs (b) and CNs (c) of Li^+^ in FTPM. d) FTIR spectra of FEC‐TFP, LE, and FTPM. e) Raman spectra of FTPM, LE, and FEC‐TFP. f) HOMO and LUMO of LiDFOB, TFP, FEC, and PFPMA, respectively.

To reveal the interaction between PFPMA and solvents, electrostatic potential (ESP) calculations were conducted (**Figure** **2**a; Figures S1 and S2, Supporting Information). Due to the strong electron‐withdrawing effect of F atoms, the center of the benzene ring in PFPMA exhibits the strongest positive potential, forming the π‐hole. The carbonyl oxygen atoms in fluoroethylene carbonate (FEC) and tris(2,2,2‐trifluoroethyl) phosphate (TFP) display negative potential. Therefore, the π‐holes on the side chains of the polymer skeleton can form a halogen bond with solvent molecules.^[^ ^23^ ^]^ The halogen bond energy was evaluated using density functional theory (DFT) calculations (Figure S3, Supporting Information). The calculated binding energies of PFPMA with solvent molecules are on the same level as those of typical hydrogen bonds and substantially surpass conventional van der Waals interactions. The halogen bonds weaken the coordination between Li^+^ and solvents, facilitating the formation of an anion‐dominated weakly solvating structure. The Li^+^ solvation structures in FTPM and control liquid electrolyte (LE, 1 m LiDFOB in FEC/TFP, 2:1 in vol) were analyzed through molecular dynamics (MD) simulation. For FTPM, the radial distribution functions (RDFs) and corresponding coordination numbers (CNs) of Li^+^ are presented in Figure 2b,c. In FTPM, the initial peak corresponding to Li^+^−O (DFOB^−^) occurs at 2.06 Å, which is much shorter than that of Li^+^−O (FEC) (2.14 Å) and Li^+^−O (TFP) (2.18 Å). At a distance of 3.0 Å, the CNs for FEC, TFP, and DFOB^−^ are 1.48, 0.28, and 2.78, respectively. These indicate the formation of an anion‐dominated weakly solvating structure in FTPM. In contrast, the Li^+^ solvation structure in LE remains solvent‐dominated. At 3.0 Å, the CNs for FEC, TFP, and DFOB^−^ are 2.46, 0.51, and 1.74, respectively (Figure S4, Supporting Information). For RDFs, the initial peak for Li^+^−O (DFOB^−^) appears at 2.07 Å, comparable to that in FTPM, whereas the initial peaks for Li^+^−O (FEC) and Li^+^−O (TFP) shift to shorter distances of 2.04 and 2.11 Å, respectively (Figure S5, Supporting Information). These demonstrate that the polymer skeleton with π‐holes effectively pulls solvent molecules out of the first Li^+^ solvation sheath.

The effect of the halogen bond on the Li^+^ solvation structure was further verified by Fourier transform infrared (FTIR) spectroscopy. As illustrated in Figure 2d, the characteristic peak of DFOB^−^ undergoes a blue shift upon incorporation of the π‐hole. This spectral change indicates the enhanced contact frequency between Li^+^ and DFOB^−^ within the solvation sheath, resulting in increased populations of contact ion pairs (CIP) and aggregate (AGG) clusters.^[^ ^24^, ^25^ ^]^ Nuclear magnetic resonance (NMR) spectroscopy was employed to further validate the evolution of the solvation structure. Upon incorporation of the polymer skeleton, the distinct low‐field shift of the ^7^Li NMR signal suggests a weakened binding environment for Li⁺ (Figure S6, Supporting Information). Meanwhile, high‐field shifts are observed for the ^11^B and ^19^F nuclei in DFOB^−^ as well as for the ^19^F nucleus in FEC. These shifts indicate an increased proportion of Li⁺‐anion pairs/aggregates and a reduced coordination between FEC and Li⁺ (Figures S7 and S8, Supporting Information).^[^ ^1^ ^]^ Raman spectra of the solvent molecules and electrolytes are presented in Figure 2e. In FEC‐TFP, the peaks observed at 866 and 906 cm^−1^ are attributed to the C─F stretching vibrations and ring skeleton deformation of FEC, respectively. The additional peak at 922 cm^−1^ in LE can be assigned to Li⁺‐solvating FEC.^[^ ^26^, ^27^ ^]^ Following incorporation of the π‐hole, the presence of Li⁺‐solvating FEC diminishes significantly. This demonstrates that the interactions between the polymer skeleton and FEC effectively attenuate Li⁺‐FEC coordination, thereby enabling more anions to enter into the first Li^+^ solvation sheath.

The diffusion coefficients of individual species in the FTPM were calculated by fitting the time‐dependent mean‐square displacement (MSD) curves to the Einstein equation (Figure S9a, Supporting Information). The diffusion coefficients of Li^+^, DFOB^−^, TFP and FEC are 1.67 × 10^−11^, 1.33 × 10^−11^, 8.33 × 10^−11^ and 1.67 × 10^−10^ m^2^ s^−1^, respectively (Figure S9b, Supporting Information). The diffusion coefficients of solvent molecules are significantly higher than those of Li^+^ and DFOB^−^ indicating that the dynamic halogen bonding between polymer and solvents does not substantially restrict the solvents mobility. Consequently, solvent molecules retain rapid transport ability along the polymer.^[^ ^28^ ^]^

The lowest unoccupied molecular orbital (LUMO) and highest occupied molecular orbital (HOMO) are critical indicators for assessing the electrochemical stability of electrolytes.^[^ ^29^ ^]^ Thus, we evaluated the decomposition tendencies of electrolyte components through DFT calculations (Figure 2f). The low LUMO of PFPMA promotes its preferential decomposition for solid electrolyte interphase (SEI) formation, inducing the generation of LiF‐rich SEIs with both high interfacial energy and superior mechanical strength.^[^ ^26^ ^]^ The LUMO of LiDFOB is comparable to that of PFPMA, suggesting the co‐formation of an SEI layer containing both LiF and Li_x_BO_y_F_z_ hybrid phases at the Li metal surface. This hybrid SEI would enhance interfacial ion conduction and improve chemical/mechanical robustness simultaneously. The HOMO of TFP (−9.55 eV) is significantly lower than those of other components, indicating superior oxidation resistance for the strong electron‐withdrawing effect of its F atoms. This characteristic enables TFP to effectively enhance the oxidative stability of the gel electrolyte. Moreover, LiDFOB and PFPMA exhibit higher HOMO energy levels, signifying their thermodynamically favored oxidative decomposition at the cathode surface. This process forms a stable cathode electrolyte interphase (CEI) layer, thereby further enhancing the high‐voltage stability.

According to above mentioned analysis, it is clear that the abundant π‐hole functional groups on the polymer skeleton form non‐bonding interactions with solvent molecules by halogen bonds. This configuration effectively displaces solvent molecules from the first Li^+^ solvation sheath without impeding their mobility, thereby facilitating an anion‐dominated weakly solvating structure. Additionally, the skeleton participates in constructing the EEIs, enhancing the interfacial ionic conduction and stability.

### 2.2 Reversibility of Li and the Structure of SEIs

**Figure 3:**
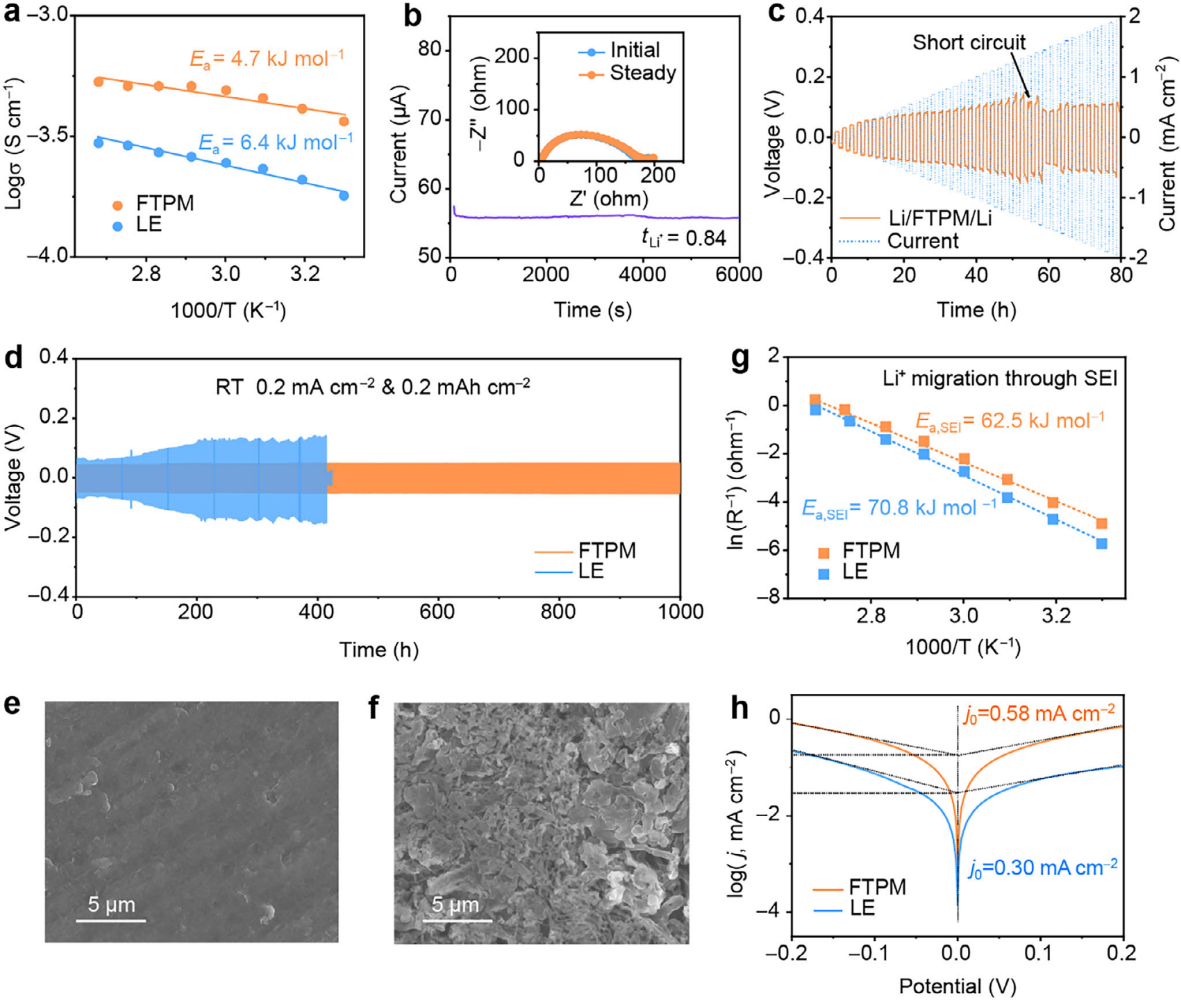
Li‐electrolytes interface stability. a) The curves of ionic conductivities with increasing temperature. b) *t*_Li+_ of FTPM at RT. c) Galvanostatic cycling curves of Li/FTPM/Li cells under increasing current densities. d) Long‐term cycling performance of Li symmetric cells. e,f) SEM images of Li from cycled Li/FTPM/Li (e) and Li/LE/Li (f). g) Arrhenius plots for the resistance of Li^+^ migration through SEI. h) Tafel plots of Li plating/stripping in different electrolytes.

To investigate the effect of polymer content on performance, we prepared a series of samples with polymer contents of 0, 5, 10, 15, and 20 wt.%, named as LE, FTPM‐5, FTPM‐10, FTPM‐15, and FTPM‐20, respectively. FTPM‐10 exhibits the highest ionic conductivity (0.30 mS cm^−1^) and the lowest interfacial impedance (Figures S10 and S11, Supporting Information). Thus, the term “FTPM” in this study specifically refers to FTPM‐10. **Figure** **3**a displays the temperature‐dependent ionic conductivity of FTPM and LE from 30 to 100 °C, which can be fitted using the Arrhenius equation. FTPM exhibits an activation energy (*E*_a_) of 4.7 kJ mol^−1^, lower than that of LE (6.4 kJ mol^−1^), suggesting reduced energy barriers for Li^+^ transport in FTPM.^[^ ^30^, ^31^ ^]^ The growth of Li dendrites is strongly correlated with the Li^+^ transference number (*t*_Li+_).^[^ ^32^ ^]^ FTPM shows a significantly higher *t*_Li+_ (0.84) compared to LE (0.44) (Figure 3b; Figure S12, Supporting Information). This enhancement may originate from a halogen bond interaction that immobilizes anions, thereby restricting their migration within the electrolyte. The interfacial stability of FTPM with Li anodes was investigated through Li symmetric cells. Gradient‐current testing revealed a critical current density (CCD) of 1.35 mA cm^−2^ for the FTPM electrolyte (Figure 3c), much higher than the LE system (0.85 mA cm^−2^) (Figure S13, Supporting Information). This demonstrates superior high‐current tolerance and stable Li plating/stripping behavior.^[^ ^33^, ^34^ ^]^ Notably, Li symmetric cells employing FTPM sustained lower overpotentials and achieved extended cycling stability exceeding 1000 h at 0.2 mA cm^−2^ and 0.2 mAh cm^−2^, whereas the LE counterparts failed after 410 h (Figure 3d). The morphologies of deposited Li metals were investigated by scanning electron microscope (SEM). The surface of Li metal after cycling in Li/FTPM/Li cells remains smooth, indicating uniform Li⁺ deposition (Figure 3e). In contrast, LE cell exhibits extensive dendritic structures and surface heterogeneity (Figure 3f). Even under a current density of 0.5 mA cm^−2^ and an area capacity of 0.5 mAh cm^−2^, a Li symmetric cell using FTPM can still achieve stable cycling for 1000 h (Figure S14, Supporting Information). These observations confirm that FTPM can effectively stabilize the Li metal anode.

The Li deposition behaviors were further investigated by the variation of interfacial resistance with temperature in Li symmetric cells (Figures S15 and S16, Supporting Information). The activation energy (*E*_a, SEI_) of Li^+^ transport through the SEI layer was significantly lower in the FTPM cell (62.5 kJ mol^−1^) than in the LE cell (70.8 kJ mol^−1^) shown in Figure 3g. This indicates that Li^+^ can pass through the SEI layer faster in FTPM, ensuring abundant Li^+^ content and uniform Li^+^ deposition under the SEI layer.^[^ ^35^ ^]^ In contrast, in LE, the higher *E*_a, SEI_ results in slow diffusion of Li^+^ through the SEI, which causes the insufficient Li^+^ below the SEI to preferentially aggregate and form dendrites. In addition, the exchange current density (*j*_0_) of Li/FTPM/Li cells is much higher than that of Li/LE/Li cells (0.58 vs 0.30 mA cm^−2^), which further confirms the fast interfacial transport kinetics in the FTPM system (Figure 3h).^[^ ^36^, ^37^ ^]^

**Figure 4:**
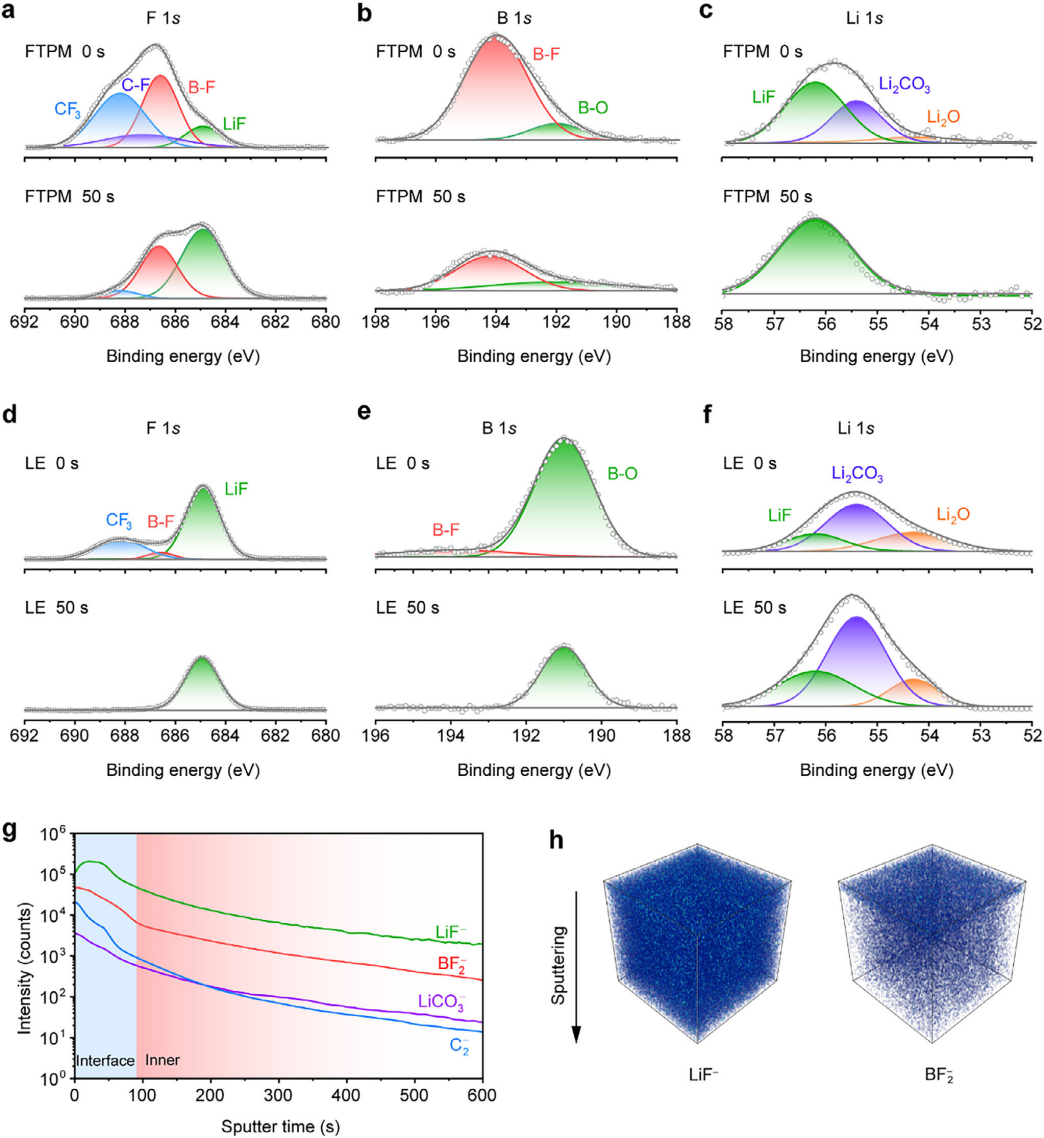
Characterization of SEI layers. a–c) XPS spectra of Li metal showing the F 1*s* (a), B 1*s* (b), and Li 1*s* (c) regions after 100 cycles in a Li/FTPM/Li cell at 0.2 mA cm^−2^ and 0.2 mAh cm^−2^. d–f) XPS spectra of Li metal showing the F 1*s* (d), B 1*s* (e), and Li 1*s* (f) regions after 100 cycles in a Li/LE/Li cell at 0.2 mA cm^−2^ and 0.2 mAh cm^−2^. g, h) TOF‐SIMS depth profiles (g) and 3D view of fragments (LiF^−^ and BF_2_^−^) distribution (h) of cycled Li metal from Li/FTPM/Li cell.

Li^+^ migration through SEI is closely related to the chemical composition of SEI, which was characterized by using in‐depth etching X‐ray photoelectron spectroscopy (XPS) and time‐of‐flight secondary ion mass spectrometry (TOF‐SIMS). The anion‐dominated weak solvation structure in FTPM promotes the formation of inorganic‐rich SEI layers, including LiF (**Figure** **4**a,c) and B and F species with low interfacial impedance (Figure 4a,b).^[^ ^12^, ^38^, ^39^ ^]^ The content of organic species (Figure S17, Supporting Information) in the inner layer of SEI is significantly reduced.^[^ ^39^, ^40^ ^]^ In contrast, the inner layer of the SEI formed by LE has a higher content of organic components and fewer LiF components, which would be caused by the decomposition of organic solvents (Figure 4d,f; Figure S18, Supporting Information). Furthermore, the B‐containing component in LE is dominated by B─O, which is attributed to the decomposition of the electrolyte and conversion of B─F into B─O bonds (Figure 4e).^[^ ^41^, ^42^ ^]^ In order to elucidate the composition and structure of the SEI formed on the Li anode after cycling, further TOF‐SIMS analysis was performed. Figure 4g shows the variation of the contents of each component with depth in the SEI formed by FTPM. From the surface to the inner layer of the SEI, the LiF and B‐F signals are much more intense than the organic components. The corresponding 3D reconstructed image also shows a homogeneous distribution of the inorganic components, whereas the signal of Li_2_CO_3_ and organic components almost disappears in the inner layer (Figure 4h; Figure S19, Supporting Information). LiF and B‐rich species are uniformly distributed in the outer layer of the SEI for FTPM battery (Figure S20, Supporting Information). In contrast, the SEI formed by LE is dominated by organic components with a relatively weak LiF signal (Figures S21 and S22, Supporting Information). Therefore, the anion‐dominated solvation structure in FTPM resulted in anion‐derived SEIs with good Li metal compatibility and fast Li^+^ conductivity, which significantly reduced the interfacial impedance of the battery and effectively suppressed Li dendrites. Even at an elevated temperature of 80 °C, FTPM still facilitates the formation of an inorganic‐dominated SEI layer, enabling homogeneous Li^+^ deposition (Figures S23 and S24, Supporting Information).

### 2.3 Oxidation Stability and Battery Performance

**Figure 5:**
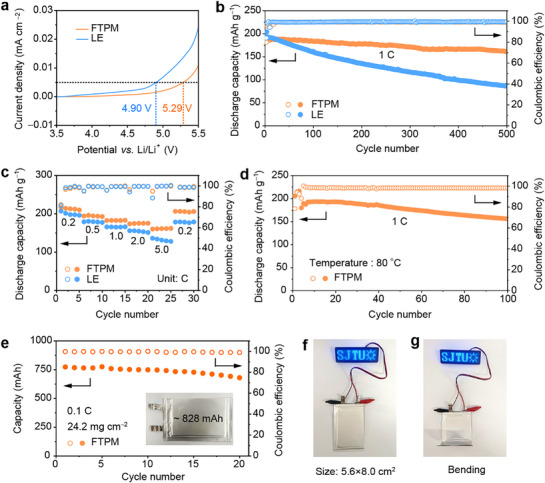
Electrochemical performance of LMBs. a) LSV curves of FTPM and LE. b) Long‐term cycling performance of Li/NCM811 batteries at RT. c) Rate performance of Li/NCM811 batteries with different electrolytes at RT. d) Cycling performance of the Li/FTPM/NCM811 battery at 80 °C. e) Cycling performance of the Li/FTPM/NCM811 pouch cell at RT. f,g) Li/FTPM/NCM811 pouch cell powering an LED board (f) and under bending (g).

After investigating Li metal compatibility, we further explored the stability of the electrolyte paired with high‐voltage cathodes. Linear scanning voltammetry (LSV) curves indicate that the polymer skeleton increases the oxidation potential from 4.90 to 5.29 V (**Figure** **5**a). A self‐discharge test was conducted to evaluate the reactivity between the electrolyte and the cathode by monitoring the open‐circuit voltage of a fully charged battery.^[^ ^43^ ^]^ For a fully charged Li/NCM811 cell (4.5 V) using FTPM, a stable voltage (≈4.2 V) is maintained even after 20 days of storage, whereas the voltage rapidly drops (<3.8 V) within 10 days for a cell using LE (Figure S25, Supporting Information). The cycling performance of the cell after self‐discharge storage is shown in Figure S26 (Supporting Information). Due to the continuous decomposition of LE, the capacity of the Li/LE/NCM811 cell fades rapidly, delivering a discharge capacity of only 83.6 mAh g^−1^ after 200 cycles at 1 C, which is merely 59.1% of that of the Li/FTPM/NCM811 cell. The above results demonstrate that FTPM exhibits excellent oxidative stability and can effectively suppress side reactions.

To further validate the cell performance, Li/NCM811 cells were assembled with FTPM and LE, respectively, and cycled at RT. The Li/FTPM/NCM811 cell (3–4.5 V, mass loading 3 mg cm^−2^) delivers 161.0 mAh g^−1^ discharge capacity with 84.5% capacity retention after 500 cycles at 1 C (Figure 5b; Figure S27, Supporting Information). In comparison, the Li/NCM811 cell with LE delivers a discharge capacity of only 86.6 mAh g^−1^, retaining only 44.7% of the initial value (Figure 5b; Figure S28, Supporting Information). Notably, the FTPM‐based Li/NCM811 cells demonstrate superior rate capability with specific capacities of 213.4, 194.4, 182.8, 174.9, 161.3, and 205.9 mAh g^−1^ at varying current densities (0.2, 0.5, 1, 2, 5, and return to 0.2 C, respectively), significantly outperforming LE counterparts (200.1, 179.1, 165.7, 154.3, 131.9, and 178.3 mAh g^−1^ under identical conditions) (Figure 5c). The Li/FTPM/NCM811 cells achieve a discharge capacity of 174.8 mAh g^−1^ with 94.2% capacity retention after 200 cycles at a high rate of 2 C (Figures S29 and S30, Supporting Information). FTPM also demonstrates compatibility with high‐voltage LiCoO_2_ (LCO) cathodes. The Li/FTPM/LCO cells exhibit superior cycling performance within an extended operating window of 3‐4.6 V (Figure S31, Supporting Information). Furthermore, FTPM mitigates the high‐temperature performance degradation of NCM811 cathodes. The Li/FTPM/NCM811 battery exhibits excellent rate performance at 80 °C, delivering an average discharge capacity of 150.7 mAh g^−1^ even at 2 C (Figure S32, Supporting Information). The Li/FTPM/NCM811 cell demonstrates a discharge capacity of up to 156.0 mAh g^−1^ after cycling 100 cycles at a 1 C and 80 °C, exhibiting an ultralow average capacity fade of 0.14% per cycle (Figure 5d). As shown in Table S1 (Supporting Information), which compares the high‐temperature performance of FTPM with other gel electrolytes, FTPM demonstrates superior high‐temperature cycling stability among current gel electrolytes for LMBs.

To evaluate the practical viability of FTPM in high‐energy‐density batteries, Li/FTPM/NCM811 pouch cells were fabricated. As shown in Figure 5e, a pouch cell with a designed capacity of 828 mAh was assembled (cathode: double‐sided coated with a mass loading of 24.2 mg cm^−2^; anode: Li foil anode with a thickness of 50 µm). After 20 cycles at 0.1 C, it exhibited a discharge capacity of 680.1 mAh. The voltage‐capacity curves during the formation process show that the as‐assembled pouch cell has an average discharge voltage of 3.78 V and a practical discharge capacity of 805.6 mAh (Figure S33, Supporting Information), corresponding to an energy density of 462.2 Wh kg^−1^ (Table S2, Supporting Information), irrespective of packaging materials. The pouch cell can power an LED board in both flat and bent states (Figure 5f,g). Nail penetration tests were performed on the pouch cell (Video S1, Supporting Information). No smoke or ignition was observed during the test. The cell voltage exhibited only a minor drop from 4.231 to 4.225 V after the test (Figure S34, Supporting Information). These results confirm the outstanding safety performance of the electrolyte, indicating its capability to maintain battery operation under severe mechanical abuse.

### 2.4 Cathode Stability and CEI Chemistry

**Figure 6:**
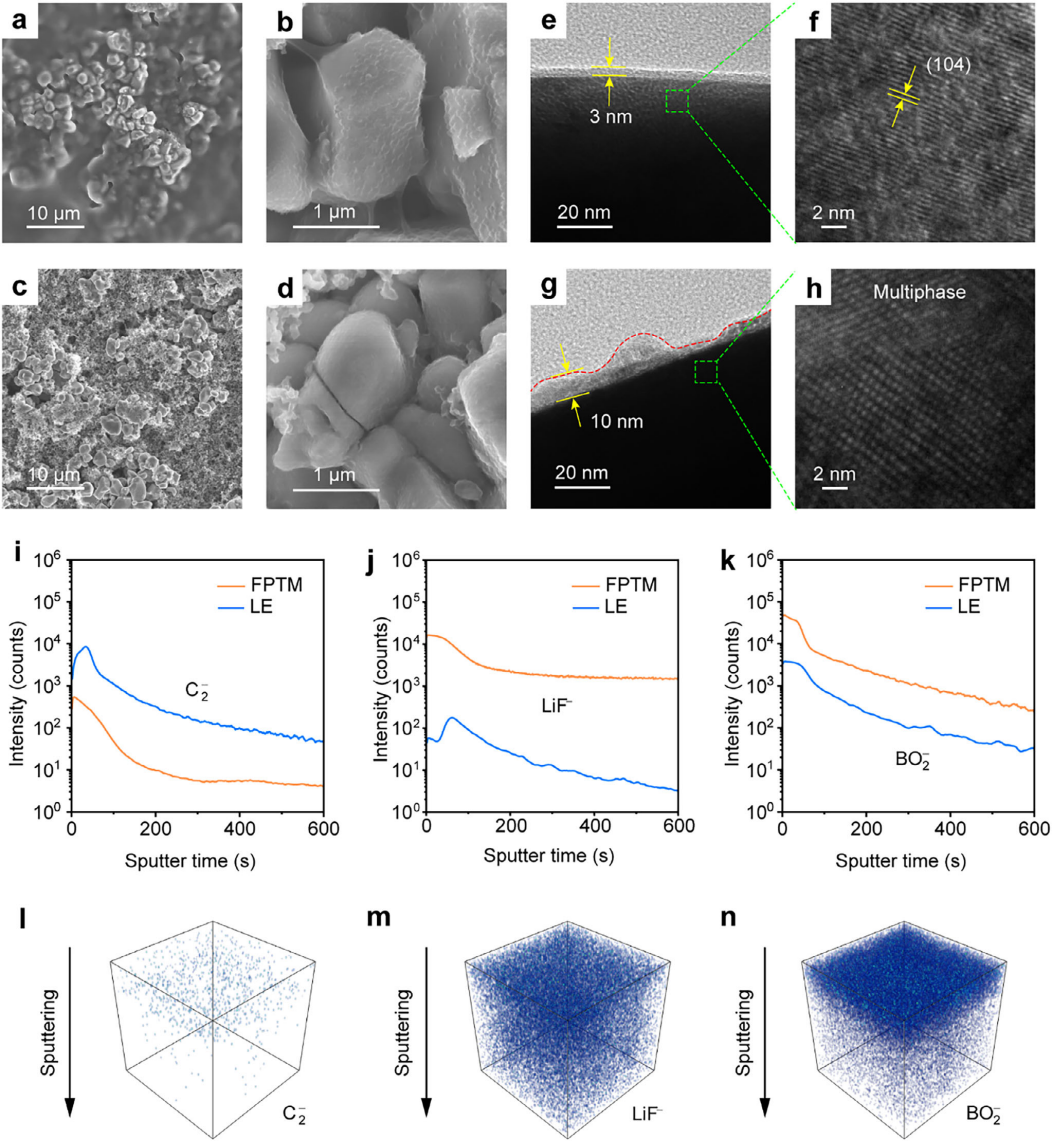
NCM811‐electrolytes interfaces characterization. a–d) SEM images of NCM811 cathodes after 100 cycles in FTPM (a,b) and LE (c,d), respectively. e–h) TEM images of NCM811 cathodes after 100 cycles in FTPM (e,f) and LE (g,h), respectively. i–k) The intensity depth profiles of TOF‐SIMS for C_2_^−^ (i), LiF^−^ (j), and BO_2_^−^ (k) fragments, respectively. l–n) The 3D views of C_2_^−^ (l), LiF^−^ (m), and BO_2_^−^ (n) fragments with FTPM, respectively.

To unravel the stability mechanism of FTPM coupled with high‐voltage cathodes, the NCM811 after 100 cycles in different electrolytes was observed by SEM. The surface of the NCM811 cathode after cycling in FTPM has an obvious polymer coating, which indicates that the small‐molecule precursor penetrates well into the inside of the cathode before thermal polymerization, forming a close contact between FTPM and cathode particles (**Figure** **6**a,b). In contrast, the surface of the NCM811 cathode after cycling in LE shows obvious holes, and some NCM811 particles are cracked (Figure 6c,d).^[^ ^41^, ^44^ ^]^ These indicate that FTPM can effectively maintain the integrity of the cathode under high voltage. The cathode phase transition is also an important factor for cell capacity degradation. The crystal structure of the cathode after cycling was characterized using high‐resolution transmission electron microscopy (HRTEM).^[^ ^45^ ^]^ As shown in Figure 6e, the cathode particles paired with FTPM form a uniformly dense thin CEI (≈3 nm), while a thick and irregular CEI is observed in the LE system (Figure 6g). Furthermore, the NCM811 particles cycled in FTPM maintain an intact layered lattice structure (Figure 6f), whereas those cycled in the LE system undergo significant phase transformation (Figure 6h). These findings demonstrate that a robust CEI is formed in the FTPM system, resulting in exceptional cycling stability for high‐voltage LMBs. In contrast, the unstable CEI in LE induces structural degradation of NCM811 and persistent electrolyte decomposition.

The composition of CEI was further analyzed by XPS. The CEI derived by FTPM exhibits a lower C atomic ratio than that in LE, indicating less organic species generated by solvent decomposition (Figure S35, Supporting Information).^[^ ^46^ ^]^ As shown in Figure S36a (Supporting Information), the obvious LiF and B‐F signals in the F1s spectrum indicate that FTPM has formed a CEI structure rich in inorganic substances.^[^ ^47^, ^48^ ^]^ The oxidative decomposition of the solvent is significantly inhibited due to the anion‐dominated weak solvation structure in FTPM, and more anions are involved in the CEI formation process. In contrast, there is little LiF in the CEI generated by LE (Figure S36b, Supporting Information). Compared with PTFM, the O 1*s* spectrum of CEI generated by LE shows obvious P─O signals, which confirms the massive decomposition of TFP molecules on the cathode surface (Figure S37, Supporting Information).^[^ ^49^ ^]^ Although LiDFOB has the highest HOMO in LE, due to the strong solvation structure in LE, solvent molecules are more likely to migrate alongside Li⁺ toward the cathode surface. Then, solvents undergo preferential oxidation and decomposition, generating organic‐dominated CEI.

The composition and derived species distribution of CEI were further revealed by TOF‐SIMS. The C_2_^−^ fragment originates from organic components derived from solvents, whereas the LiF^−^ and BO_2_^−^ fragments correspond to the inorganic species LiF and lithium borates (LiB_x_O_y_), respectively.^[^ ^50^, ^51^, ^52^ ^]^ Compared to LE, the CEI generated by PTFM exhibits lower C_2_^−^ signal intensity with higher LiF^−^ and BO_2_^−^ signal intensities (Figure 6i–k). The 3D reconstruction images of these components in the PTFM‐generated CEI reveal localized enrichment of LiB_x_O_y_ species at the CEI surface, while the entire CEI layer demonstrates minimal organic species and a high content of LiF (Figure 6l–n). These results indicate that PTFM promotes the formation of an inorganic‐rich CEI layer on the cathode surface, characterized by an outer LiB_x_O_y_/LiF‐rich layer and an inner LiF‐rich layer, thereby significantly enhancing the ionic conductivity, mechanical strength, and thermal stability of the interphase.^[^ ^53^ ^]^ Upon cycling at 80 °C, the FTPM electrolyte facilitates the formation of a dual‐layer CEI, characterized by an organic‐rich outer layer and an inorganic‐rich inner layer (Figure S38, Supporting Information). In these cells, the surface of the NCM811 cathode exhibits substantial polymer residues, while the primary particles maintain their structural integrity without significant cracking (Figure S39, Supporting Information). The resulting CEI has a thickness of ≈8 nm and demonstrates significantly improved homogeneity compared to that in liquid cells cycled at RT (Figure S40, Supporting Information).

## 3 Conclusion

In summary, a “mortise‐tenon joint” inspired weakly solvated gel electrolyte was successfully fabricated based on “π‐hole” functional groups on the polymer side chains, which can form dynamic non‐covalent halogen bonding with carbonyl oxygen atoms on solvent molecules. This effectively weakens solvent coordination to Li⁺, thereby enabling greater anion entry into the first Li⁺ solvation sheath. The halogen bonding‐induced weakly solvating structure facilitates the formation of inorganic‐rich EEIs, thereby endowing the electrolyte to show exceptional compatibility toward Li metal anodes and stability against high‐voltage cathodes. The Li/NCM811 cells deliver 84.5% capacity retention after 500 cycles at 1 C, and 94.2% retention after 200 cycles at 2 C. The pouch cell can achieve an energy density as high as 462.2 Wh kg^−1^. This work presents a novel strategy for regulating solvation structures and enhancing performance in gel electrolytes, offering a promising approach to developing electrolytes for high‐safety, high‐energy‐density LMBs.

## Conflict of Interest

The authors declare no conflict of interest.

## Supporting information



Supporting Information

Supplemental Video 1

## Data Availability

The data that support the findings of this study are available from the corresponding author upon reasonable request.
