# Supplementary material for: Mortise‐Tenon Joint Inspired Weakly Solvated Gel Electrolyte Based on Halogen Bonds for High‐Voltage Lithium Metal Batteries
Source: Adv Sci (Weinh). 2025 Nov 16;13(7):e18448. doi: 10.1002/advs.202518448 (PMC12866769; doi:10.1002/advs.202518448)
Supplement: Supplementary file 1 — Supporting Information [file ADVS-13-e18448-s002.docx]

Supporting Information

**Mortise-Tenon Joint Inspired Weakly Solvated Gel Electrolyte based on Halogen Bonds for High-Voltage Lithium Metal Batteries**

*Shuohan Liu,^1^ Wensheng Tian,^2^ Hui Pan,^1*^ Shunwei Chen,^3^ Xiujun Han,^3*^ Hengdao Quan,^4*^ and Shenmin Zhu^1*^*

S. Liu, H. Pan, S. Zhu

State Key Laboratory of Metal Matrix Composites, School of Materials Science and Engineering, Shanghai Jiao Tong University, Shanghai 200240, China

E-mail: [panhui115@sjtu.edu.cn](mailto:panhui115@sjtu.edu.cn); [smzhu@sjtu.edu.cn](mailto:smzhu@sjtu.edu.cn)

W. Tian

State Key Laboratory of Space Power-Sources, Shanghai Institute of Space Power-Sources, Shanghai 200245, China

S. Chen, X. Han

School of Materials Science and Engineering, Qilu University of Technology (Shandong Academy of Sciences), Jinan, Shandong 250353, China

E-mail: [xjhan@qlu.edu.cn](mailto:xjhan@qlu.edu.cn)

H. Quan

School of Chemistry and Chemical Engineering, Beijing Institute of Technology, 5 South Zhong guan cun Street, Beijing 100081, China

E-mail: [quanhengdao@bit.edu.cn](mailto:quanhengdao@bit.edu.cn)

**Experimental Section**

**Preparation of FTPM and LE**

Lithium difluoro(oxalato)borate (LiDFOB, 99.9%, Canrd New Energy Technology), tris(2,2,2-trifluoroethyl) phosphate (TFP, 98%, Adamas), fluoroethylene carbonate (FEC, 99.95%, Canrd New Energy Technology), pentafluorophenyl methacrylate (PFPMA, $\geq$97%, Aladdin) and *N*,*N*’-methylenebisacrylamide (MBA, $\geq$99%, Aladdin) were used as received. The preparation processes were performed in an argon-filled glovebox (M. Braun). To prepare the LE, 1 M LiDFOB was dissolved in FEC/TFP (2:1 in vol) solvent. For the FTPM, 10 wt.% PFPMA monomer, 1 wt.% MBA crosslinker and 0.5 wt.% 2,2’-azobis(2-methylpropionitrile) (AIBN, Aladdin) initiator were co-dissolved in LE as precursor. After that, the precursor was cured at 60 ℃ for 10 h.

**Battery Assembly**

The NCM811 cathode was prepared by blending 80 wt.% NCM811 active material (MTI), 10 wt.% polyvinylidene fluoride (PVDF, Aladdin) binder and 10 wt.% conductive carbon black (SP, MTI) in *N*-methyl pyrrolidone (NMP, Canrd) to form a uniform slurry. The slurry was evenly coated on aluminum foil and then vacuum dried at 110 ℃ for 12 h. The dried electrode was then cut into slices for further use. The mass loading of NCM811 cathode was approximately 3 mg cm⁻^2^. LCO cathodes were prepared by the same method. Coin cells were assembled in an argon-filled glovebox using Celgard 2400 separator. The precursor solution was injected into the separator, and coin cells were subsequently sealed. After resting for 1 h, the cells were thermally cured in an oven at 60 ℃ for 10 h. The pouch cell was fabricated with cathode (2 layers) and anode (3 layers) sheets and filled with electrolyte (2 g Ah^−1^). After aging for 10 h at 60 ℃, the pouch cell was activated by one initial cycle, followed by degassing.

**Electrochemical Measurements**

The ionic conductivity of the electrolytes was determined via electrochemical impedance spectroscopy (EIS) using Li symmetric cells on a Biologic VMP3 electrochemical workstation. Measurements were performed at frequencies ranging from 1 MHz to 0.1 Hz with an alternating potential amplitude of 5 mV. The ionic conductivity ($\sigma$) was calculated according to the equation:

$$\text{σ=}\frac{\text{L}}{\text{R×S}}$$

where *L* stands for the thickness of the electrolyte, *R* represents the bulk resistance of the electrolyte, and *S* is the area of the electrode. Linear sweep voltammetry (LSV) test was performed on an electrochemical station (Biologic VMP3) using Li/SS cells from 0 to 6 V with a scanning rate of 1 mV s^–1^. The oxidation current density was set as 5 μA cm^−2^ and the potential values were recorded. The Li^+^ transference number ($t_{\mathrm{Li}^{+}}$) was determined via Li symmetric cells by EIS testing and direct current (DC) polarization. $t_{\mathrm{Li}^{+}}$ is calculated by the following equation:

$$t_{\mathrm{Li}^{+}}=\frac{I_{s}\left( \Delta V-I_{0}R_{0} \right)}{I_{0}\left( \Delta V-I_{s}R_{s} \right)}$$

where $I_{s}$ and $I_{0}$ represent the steady-state and initial currents, respectively; $R_{0}$ and $R_{s}$ represent the interface resistances before and after polarization, respectively. The exchange current density was derived from Tafel analysis conducted on Li symmetric cells, scanned from −0.2 V to 0.2 V at 1 mV s⁻^1^.

The assembled Li symmetric cells and Li/NCM811 and Li/LCO cells were tested on LAND CT2001A battery testing system. Galvanostatic rate measurements were carried out from 0.05 to 2 mA cm^−2^ by using Li symmetric cells which cycled 1 time at each current density. Long-term galvanostatic cycling was performed at 0.2 mA cm^−2^ and 0.2 mAh cm^−2^ or 0.5 mA cm^−2^ and 0.5 mAh cm^−2^. The Li/NCM811 cells were cycled in a voltage range of 3-4.5 V (1C = 200 mAh g⁻^1^), and Li/LCO cells were cycled in a voltage range of 3-4.6 V (1C = 220 mAh g⁻^1^).

**Sample Characterization**

Fourier transform infrared (FTIR) spectra were collected on a Thermo Scientific Nicolet 6700 instrument. The samples were dissolved in DMSO-d6 for NMR analysis on a Bruker AVANCE III 400 MHz. Raman spectra were collected on a Via Renishaw Raman spectrometer using a 532 nm wavelength laser. The cycled batteries were disassembled in a glovebox, and the cycled Li anodes and NCM811 cathodes were washed with dimethyl carbonate (DMC, 99%, Aladdin) and dried at RT. The morphologies of Li and NCM811 were observed by scanning electron microscope (SEM, Hitachi S-4800). X-ray photoelectron spectroscopy (XPS) was performed on Thermo Scientific K-Alpha. Time-of-flight secondary ion mass spectrometry (TOF-SIMS) was carried out on a TOF-SIMS 5-100 instrument (ION-TOF GmbH) equipped with Bi^3+^ as the primary ion source (30 keV) over an area of 250 $\mu m$× 250 $\mu m$. The depth profiles of TOF-SIMS were acquired on the area of 70 $\mu m$ × 70 $\mu m$ using Cs^+^ as the sputter source (2 keV). For high-resolution transmission electron microscopy (HRTEM, JEM-2100F), the NCM811 powders scraped from the electrodes were ultrasonically dispersed in ethanol (Aladdin) and subsequently drop-cast onto a copper grid to prepare the sample. For the nail penetration test, the pouch cell was penetrated by a 3 mm diameter stainless steel nail at a speed of 30 mm min^−1^.

**Computational Methods**

The structures of FEC, TFP, PFPMA and LiDFOB were fully optimized at the ωB97XD/6-31G (d, p) level of theory by Gaussian 16.^[1,2]^ The molecular dynamics simulations were conducted by Gromacs (version 2020.6) software, adopting the OPLS-AA force field with CM5 atomic charge.^[3^⁻^6]^ After an energy minimization and a canonical ensemble (NVT) simulation at 300K for 0.5 ns, the system was equilibrated for 20 ns in the isothermal-isobaric (NPT) ensemble. An additional 20 ns NPT simulation was performed for statistics. During simulations the timestep was set as 0.5 fs, and the Newton’s equation of motion was integrated with the leap-frog algorithm. The temperature was maintained at 300 K using the stochastic velocity rescaling algorithm with a coupling time of 0.1 ps.^[7]^ The pressure was regulated to 1 atm using Parrinello-Raman algorithm with a coupling time of 0.5 ps. The fast smooth Particle-Mesh Ewald method was utilized to handle the electrostatics, where the direct space part was cutoff at 1.2 nm and the reciprocal part was performed with Fast Fourier Transformations using a grid spacing of 0.12 nm and a cubic interpolation. The Van der Waals interaction was treated using a cut-off of 1.2 nm.

The electrostatic potentials (ESPs) were calculated using the DNP 4.4 basis set and the B3LYP functional, with energy and force convergence criteria set to 10⁻^5^ Ha and 0.002 Ha Å⁻^1^, respectively.


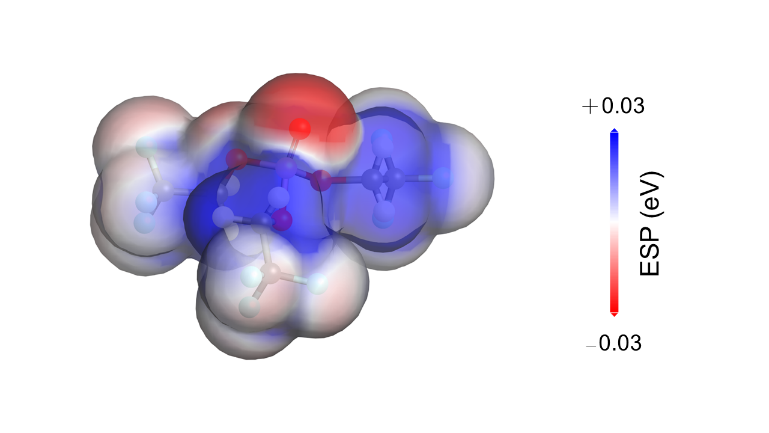


Figure S1. The ESP mapping of TFP.


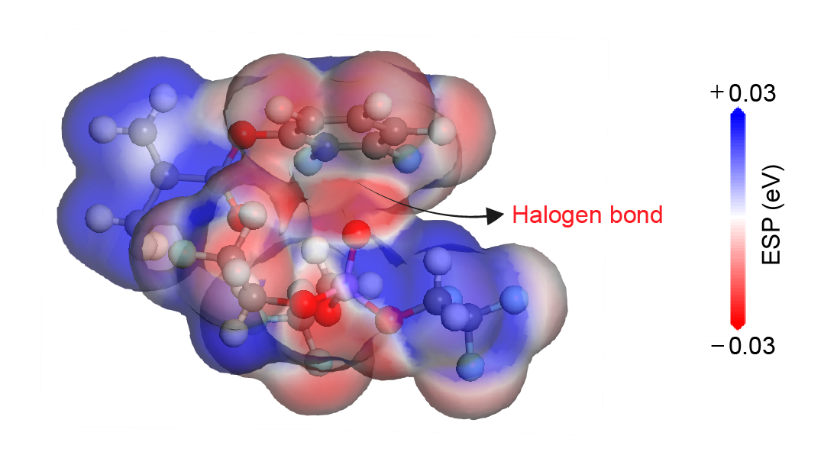


Figure S2. The ESP mapping of PFPMA-TFP.


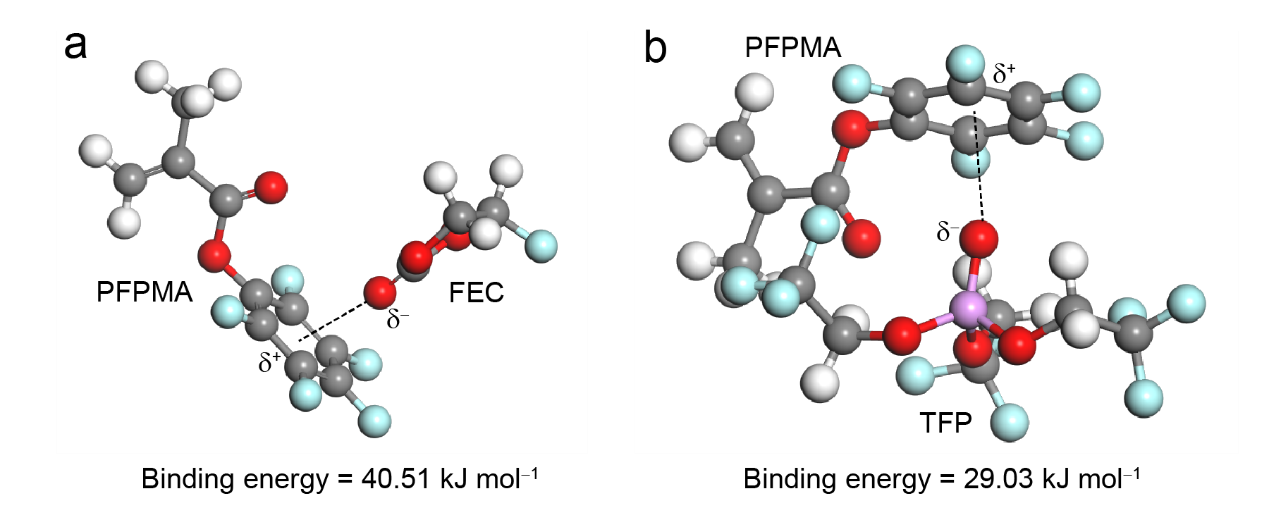


Figure S3. (a, b) Binding energies between PFPMA and FEC (a), as well as between PFPMA and TFP (b).

Figure S4. CNs of Li^+^ in LE.

Figure S5. RDFs of Li^+^ in LE.

Figure S6. ^7^Li NMR spectra of LE and FTPM.


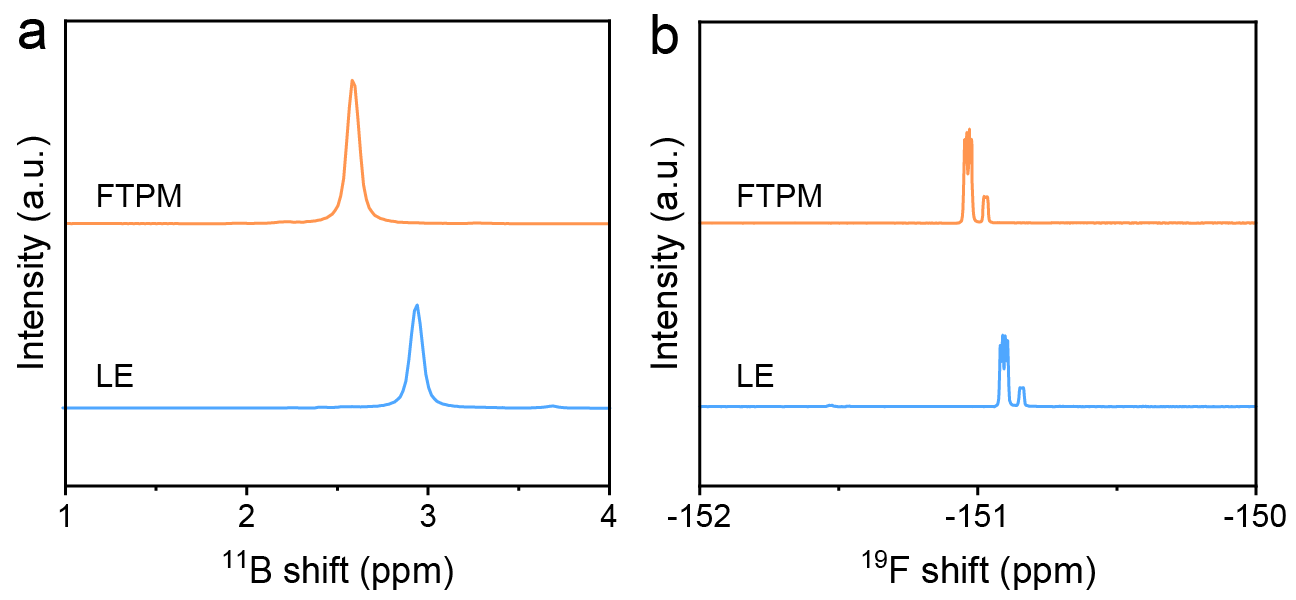


Figure S7. (a, b) ^11^B (a) and ^19^F (b) NMR spectra of DFOB anion for LE and FTPM.

Figure S8. ^19^F NMR spectra of FEC for LE and FTPM.


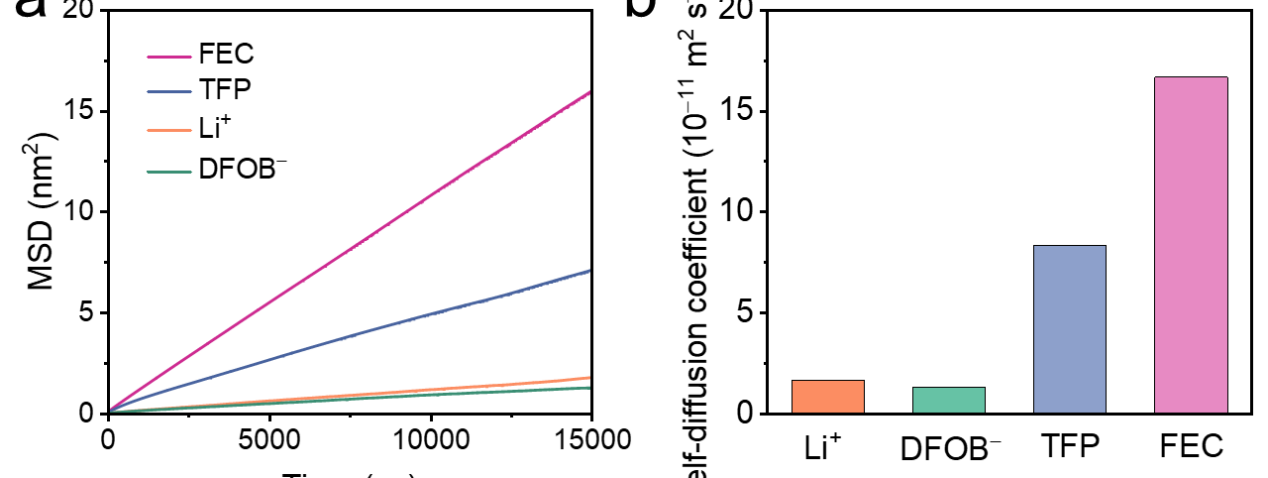


Figure S9. (a, b) MSD-time curves (a) and the corresponding diffusion coefficients (b) of FTPM components.


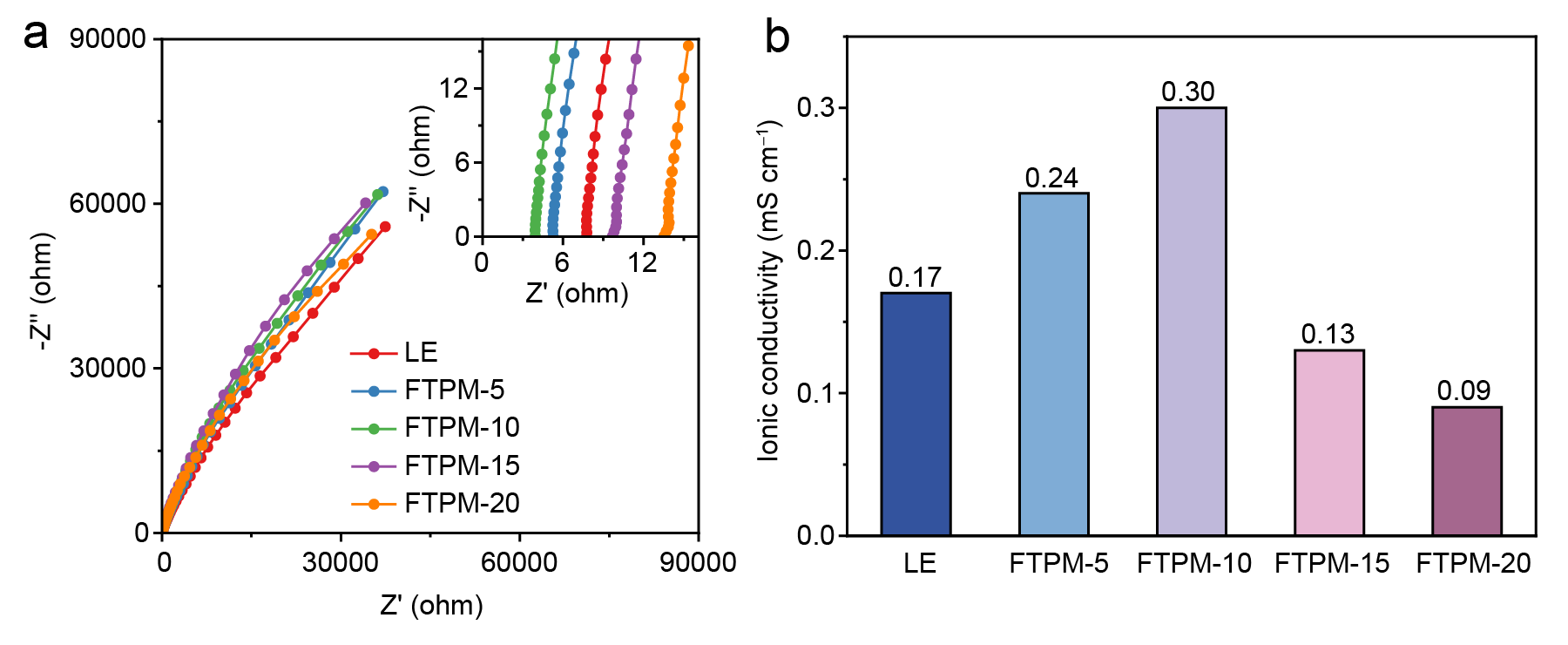


Figure S10. (a, b) Nyquist plots (a) and the corresponding ionic conductivities (b) of different electrolytes.


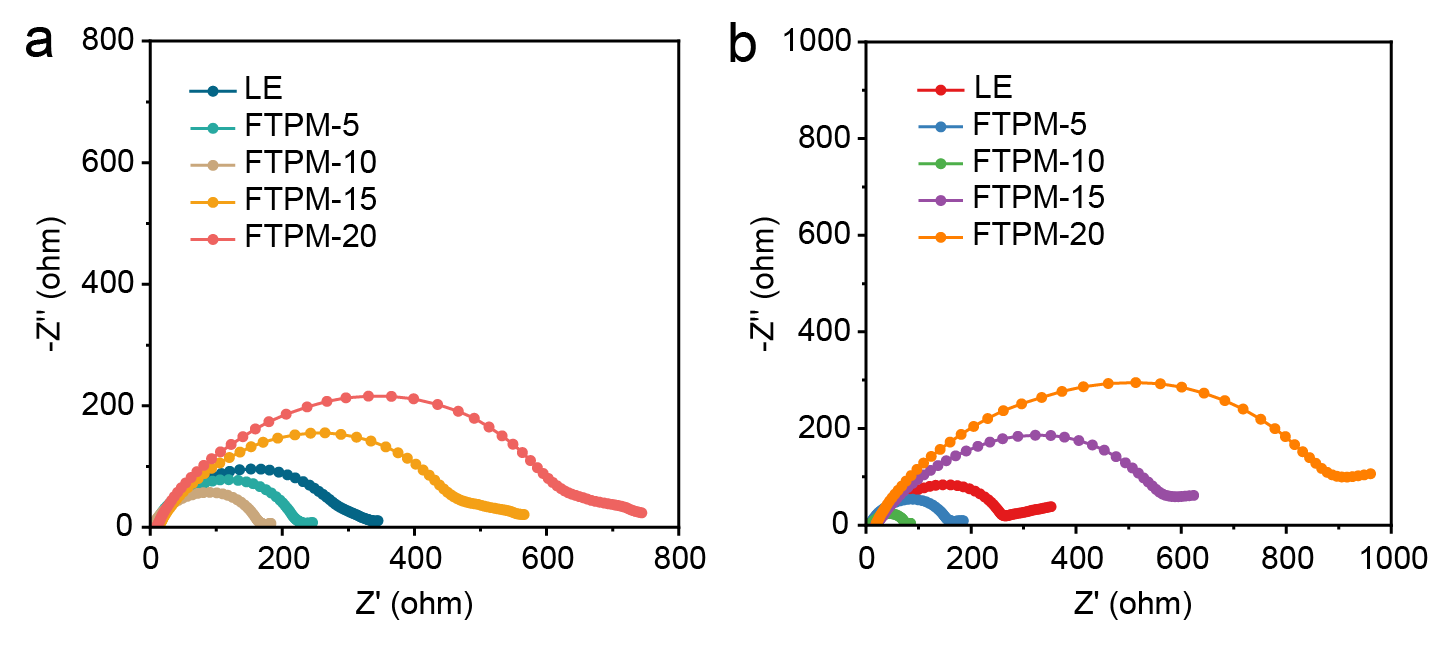


Figure S11. (a, b) Nyquist plots of Li symmetric cells with different electrolytes before (a) and after 20 cycles (b).

Figure S12. Li^+^ transference number of LE at RT.

Figure S13. Galvanostatic cycling curves of Li/LE/Li cells under increasing current densities.

Figure S14. Long-term cycling performance of the Li/FTPM/Li cell at 0.5 mA cm^−2^ and 0.5 mAh cm^−2^.

Figure S15. Nyquist impedance plots of Li/FTPM/Li cells at different temperatures.

Figure S16. Nyquist impedance plots of Li/LE/Li cells at different temperatures.

Figure S17. XPS depth profiles of C 1*s* on the Li surface after 100 cycles of Li/FTPM/Li cells at 0.2 mA cm^−2^ and 0.2 mAh cm^−2^.

Figure S18. XPS depth profiles of C 1*s* on the Li surface after 100 cycles of Li/LE/Li cells at 0.2 mA cm^−2^ and 0.2 mAh cm^−2^.


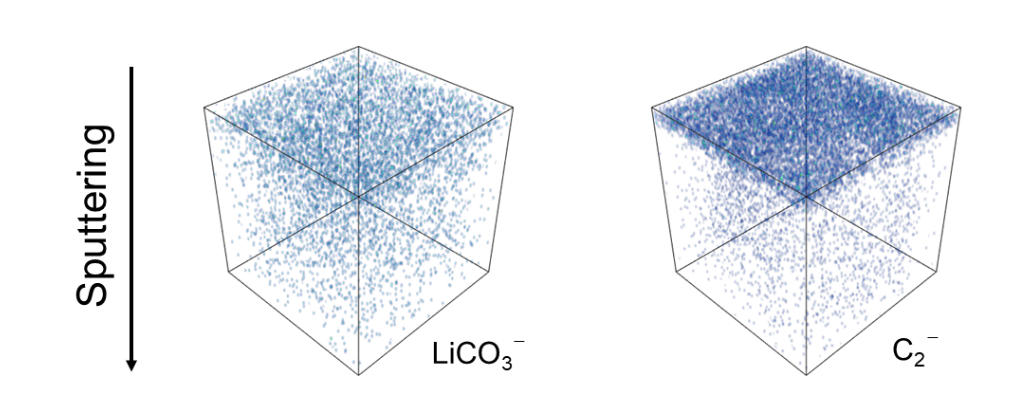


Figure S19. 3D views of LiCO_3_^−^ and C_2_^−^ distribution at the FTPM/Li interface.


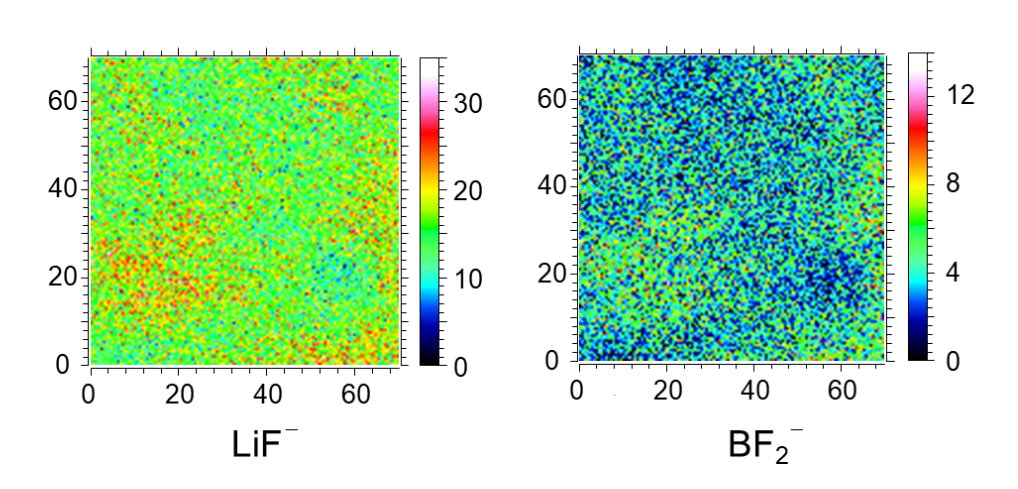


Figure S20. LiF^−^ and BF_2_^−^ fragments on the surface of Li from cycled Li/FTPM/Li cell.

Figure S21. TOF-SIMS depth profiles of various fragments formed at LE/Li interphase.


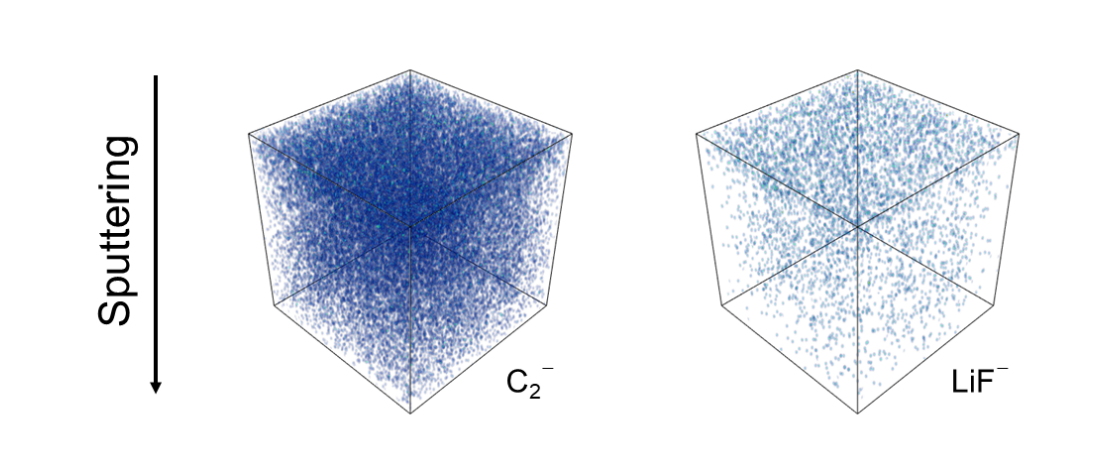


Figure S22. 3D views of C_2_^−^ and LiF^−^ distribution at the LE/Li interface.


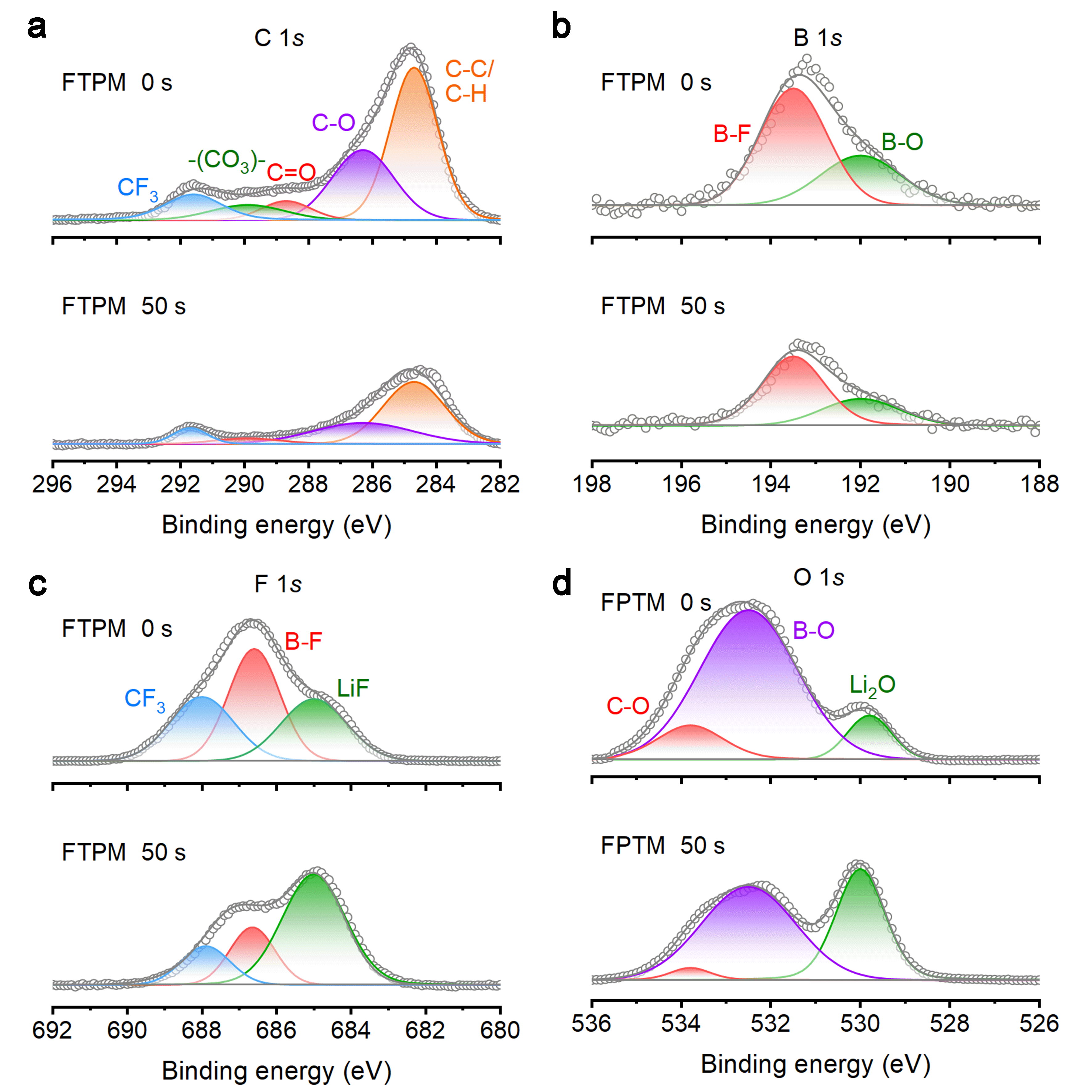


Figure S23. (a-d) XPS spectra of Li metal showing the C 1*s* (a), B 1*s* (b), F 1*s* (c) and O 1*s* (d) regions after 100 cycles in a Li/FTPM/Li cell at 80 °C.


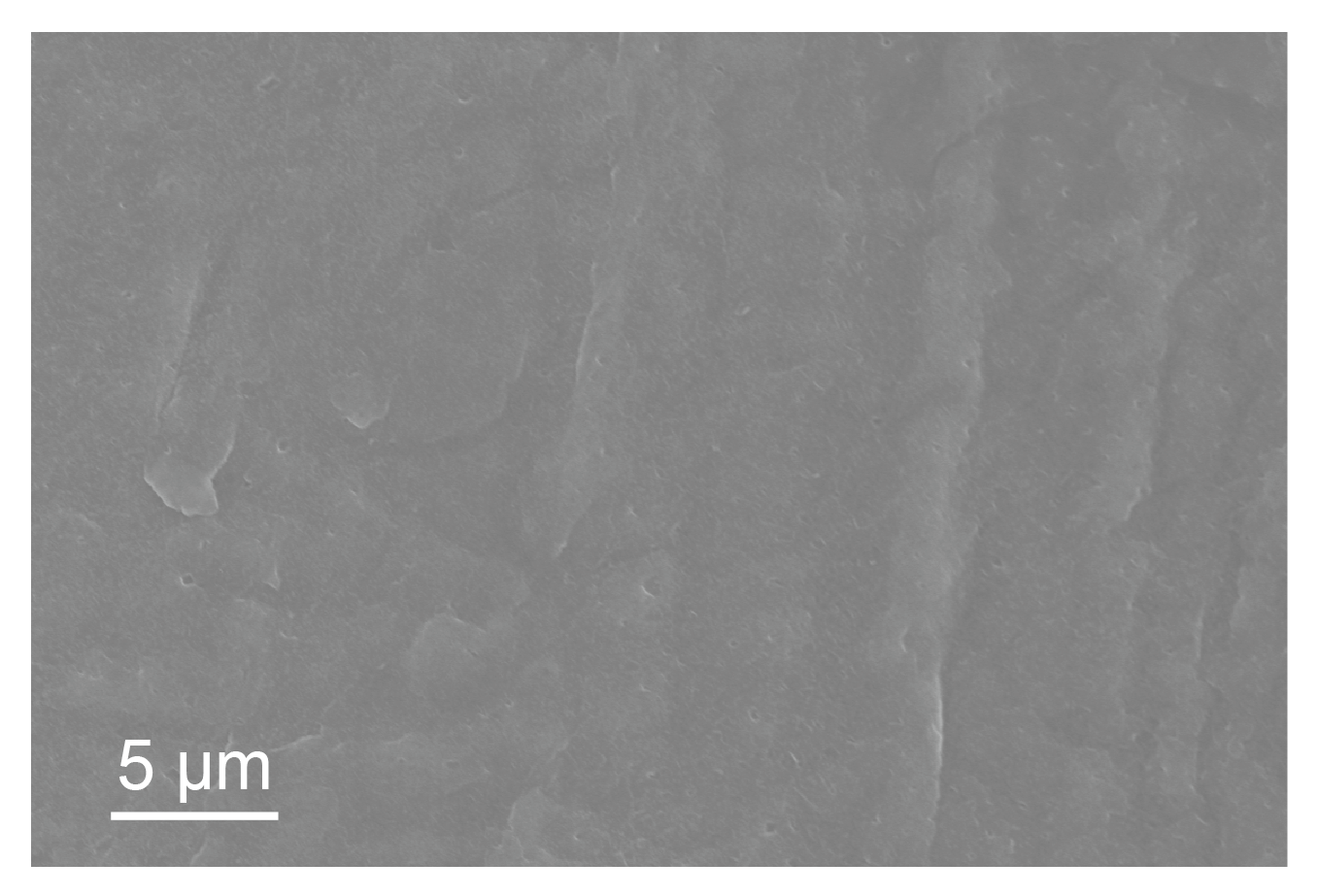


Figure S24. SEM image of Li after 100 cycles in a Li/FTPM/Li cell at 80 °C.

Figure S25. Self-discharge curves of 4.5 V fully-charged Li/NCM811 cells for 20-day storage under RT using various electrolytes.

Figure S26. Cycling performance at 1 C after 20-day self-discharge storage.

Figure S27. Charge and discharge profiles of the Li/FTPM/NCM811 battery at 1 C.

Figure S28. Charge and discharge profiles of the Li/LE/NCM811 battery at 1 C.

Figure S29. Long-term cycling performance of the Li/FTPM/NCM811 battery at 2 C.

Figure S30. Charge and discharge profiles of the Li/FTPM/NCM811 battery at 2 C.

Figure S31. Long-term cycling performance of Li/LCO batteries at 1 C.

Figure S32. Rate performance of the Li/FTPM/NCM811 battery at 80 ℃.

Table S1. Comparison of the high-temperature cycling stability of FTPM and other gel electrolytes for LMBs.

| **Electrolyte** | **Cell type** | **Capacity retention** | **Temperature** | **Refs.** |
| --- | --- | --- | --- | --- |
| L-AGPE | Li/NCM811  (3−4.3 V) | 77.5%@100 cycles@2 C | 70 ℃ | ^[8]^ |
| mSIGE | Li/NCM811  (2.8−4.2 V) | 67.5%@102 cycles@0.33 C | 55 ℃ | ^[9]^ |
| PNDA | Li/NCM811  (3−4.5 V) | 71.9%@200 cycles@1 C | 70 ℃ | ^[10]^ |
| PVC/TEP | Li/LiFePO_4_  (2.5−4.2 V) | 93%@120 cycles@0.5 C | 80 ℃ | ^[11]^ |
| E-QSPE | Li/LiFePO_4_ | 80%@500 cycles@2 C | 60 ℃ | ^[12]^ |
| DSPE | Li/LiFePO_4_ (2.5−4.2 V) | 92.9%@300 cycles@1 C | 70 ℃ | ^[13]^ |
| This work | Li/NCM811 (3−4.5 V) | 86.6%@100cycles@1 C | 80 ℃ |  |

Figure S33. The formation cycle of the Li/FTPM/NCM811 pouch cell.

Table S2. Parameters for calculation of the specific energy of the Li/FTPM/NCM811 pouch cell.

|  | Parameters | Areal weight (mg cm­^−2^) | Layers | Areal (cm^2^) | Weight (mg) |
| --- | --- | --- | --- | --- | --- |
| Li anode | 50 μm | 2.67 | 3 | 44.8 | 358.85 |
| NCM811 cathode | 70 μm | 24.2 | 2 |  | 4336.64 |
| Separator | 16 μm | 1.32 | 4 |  | 236.54 |
| Electrolyte | 2 g Ah^−1^ | | | | 1656 |
| Total weight (mg) | | | | | 6588.03 |


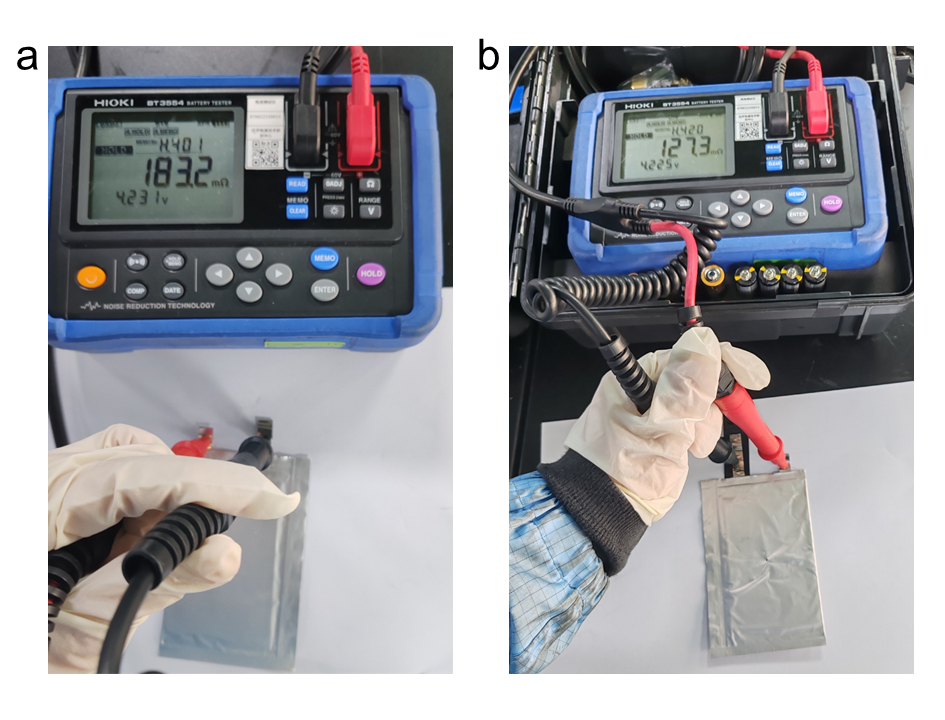


Figure S34. (a, b) The voltage of the pouch cell before (a) and after (b) needling.


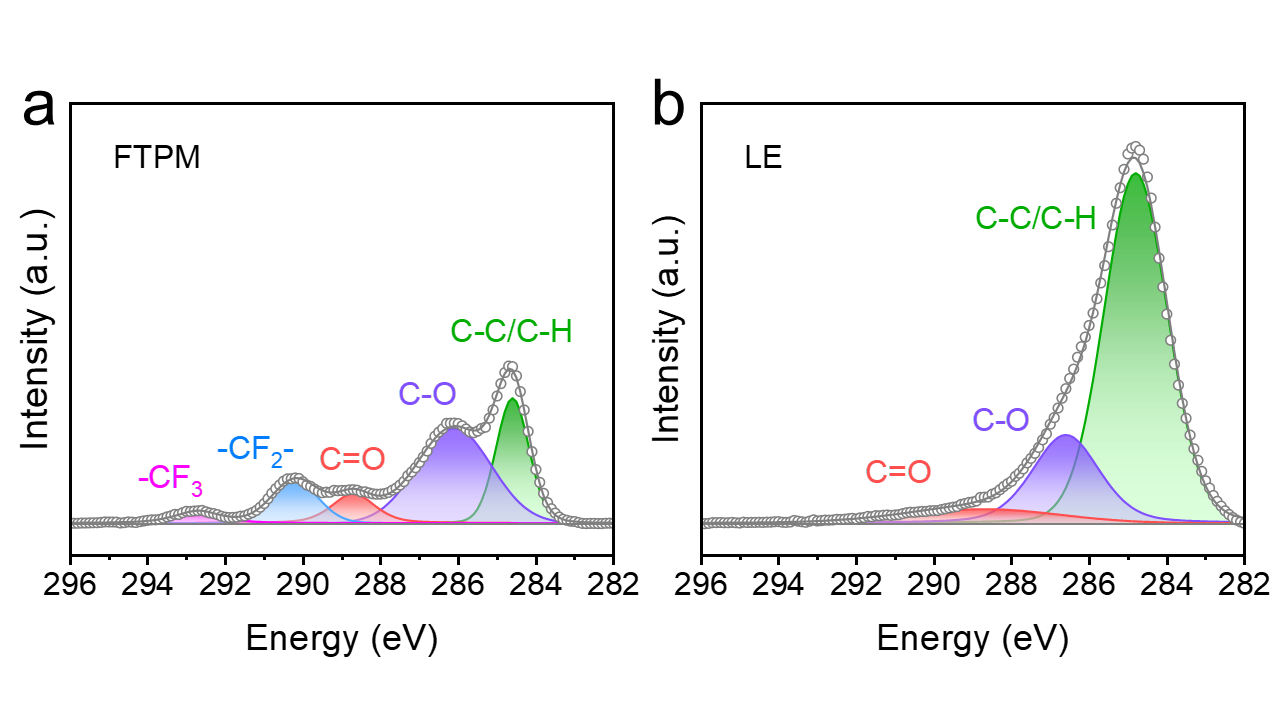


Figure S35. (a, b) XPS spectra of C 1*s* of cycled NCM811 cathode in FTPM (a) and LE (b).


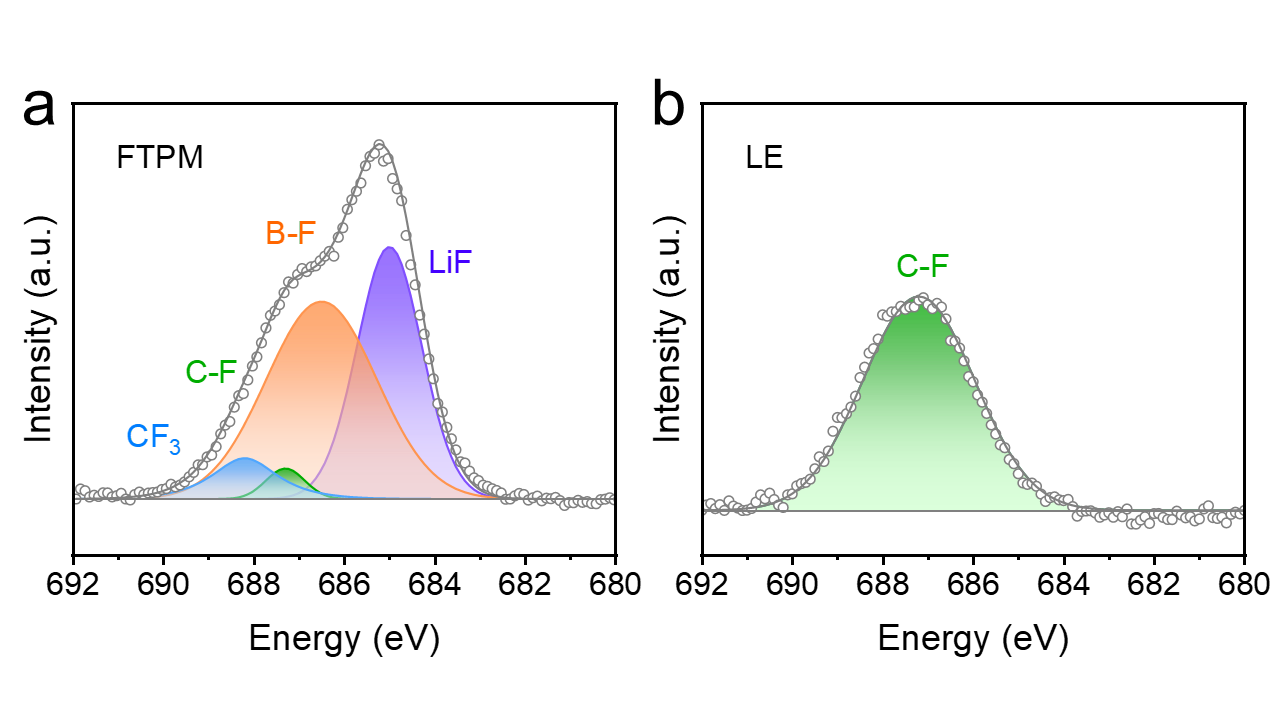


Figure S36. (a, b) XPS spectra of F 1*s* of cycled NCM811 cathode in FTPM (a) and LE (b).


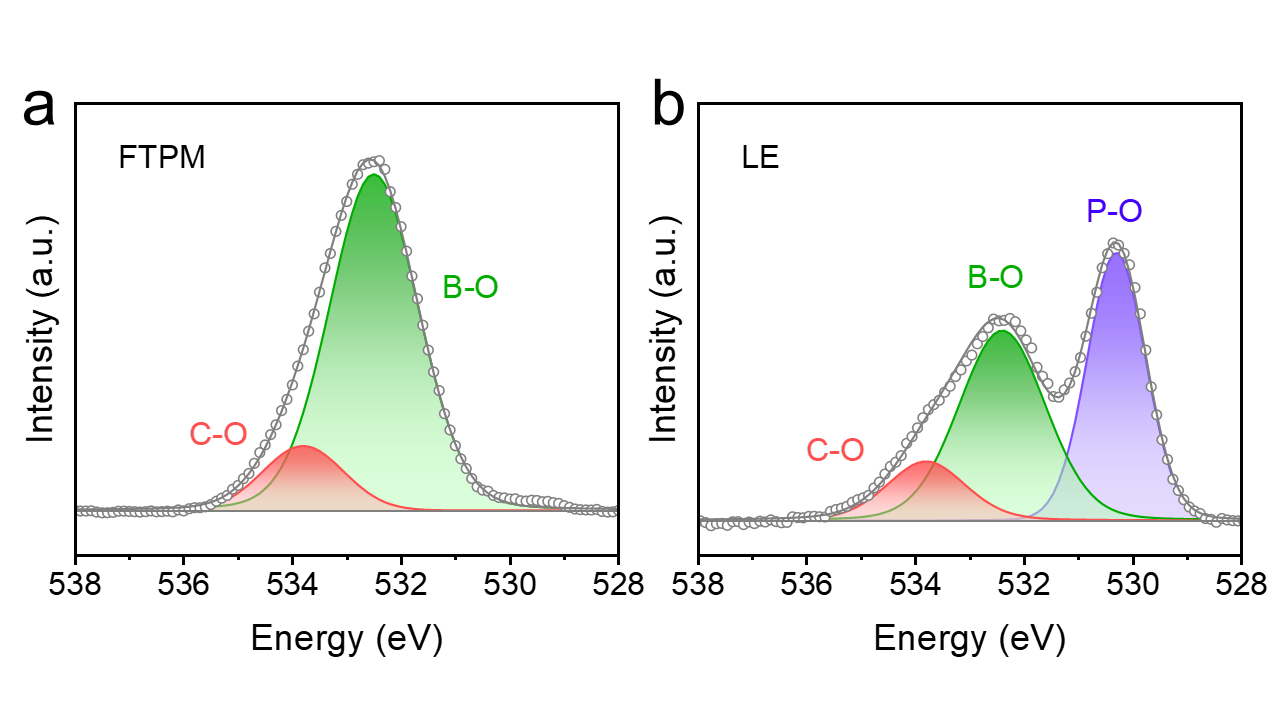


Figure S37. (a, b) XPS spectra of O 1*s* of cycled NCM811 cathode in FTPM (a) and LE (b).


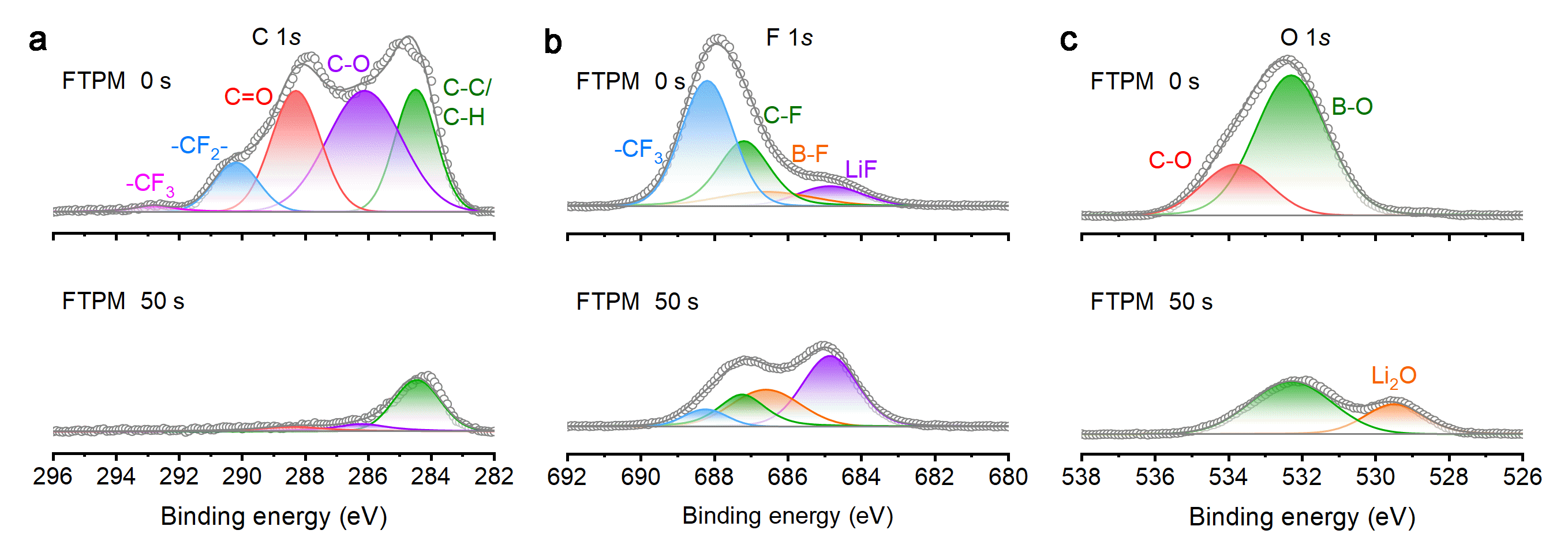


Figure S38. (a-c) XPS spectra of NCM811 showing the C 1*s* (a), F 1*s* (b) and O 1*s* (c) regions after 100 cycles in a Li/FTPM/NCM811 cell at 1C and 80 °C.


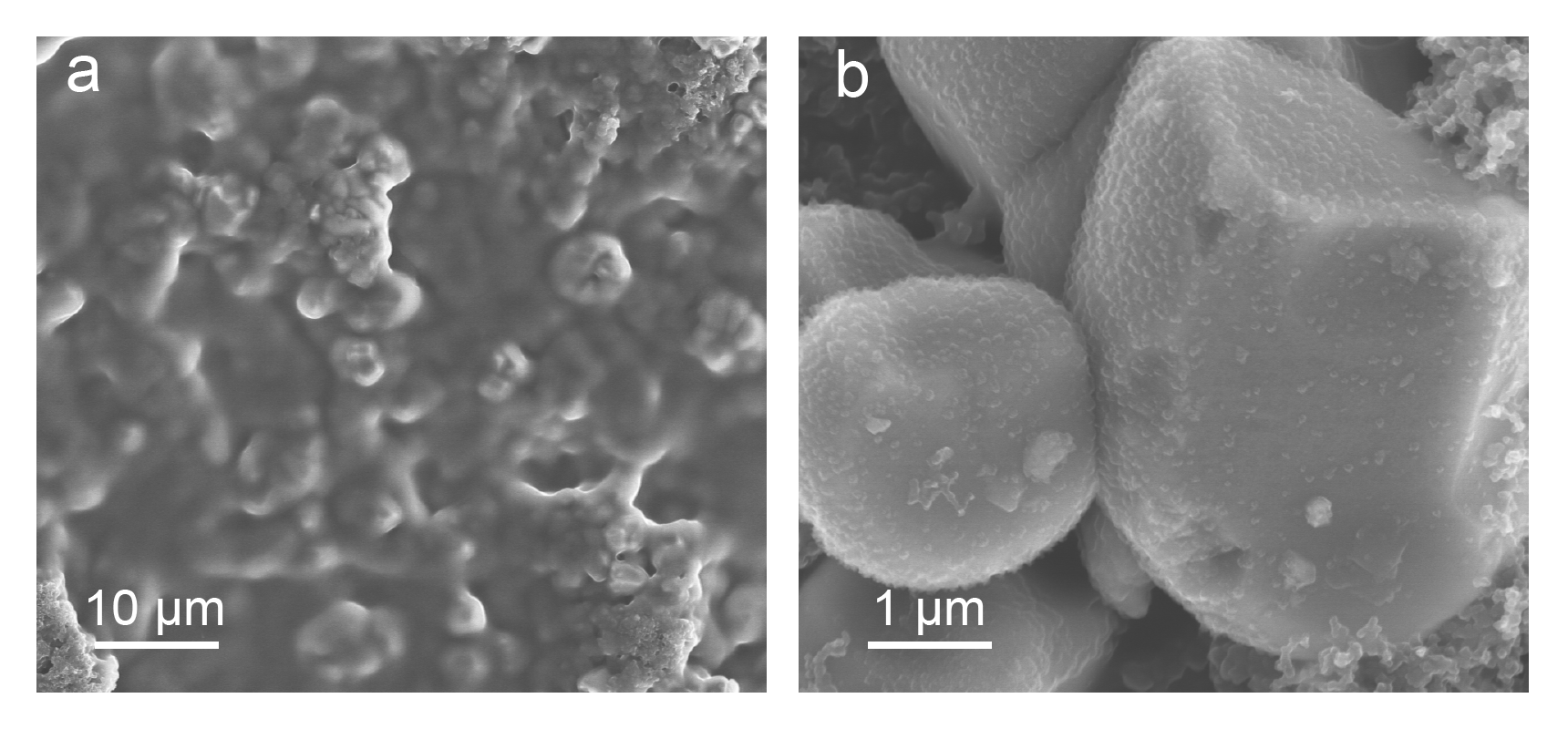


Figure S39. (a, b) SEM images of NCM811 cathodes after 100 cycles in FTPM at 1C and 80 °C.


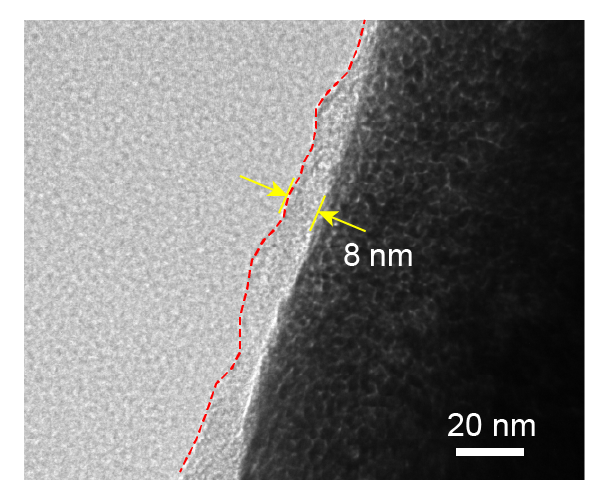


Figure S40. TEM image of NCM811 cathode cycled in FTPM electrolyte for 100 cycles at 80 °C.

**References**

[1] MJ. Frisch, GW. Trucks, HB. Schlegel, et al., “Gaussian 16,” (2016).

[2] N. Mardirossian, and M. Head-Gordon, “Thirty Years of Density Functional Theory in Computational Chemistry: An Overview and Extensive Assessment of 200 Density Functionals,” *Molecular physics* 115, no. 19 (2017): 2315-2372.

[3] D. Van Der Spoel, E. Lindahl, B. Hess, et al., “GROMACS: Fast, Flexible, and Free,” *Journal of computational chemistry* 26, no. 16 (2005): 1701-1718.

[4] GA. Kaminski, RA. Friesner, J. Tirado-Rives, and WL. Jorgensen, “Evaluation and Reparametrization of the OPLS-AA Force Field for Proteins via Comparison with Accurate Quantum Chemical Calculations on Peptides,” *The Journal of Physical Chemistry B* 105, no. 28 (2001): 6474-6487.

[5] WL. Jorgensen, DS. Maxwell, and J. Tirado-Rives, “Development and Testing of the OPLS All-Atom Force Field on Conformational Energetics and Properties of Organic Liquids,” *Journal of the american chemical society* 118, no. 45 (1996): 11225-11236.

[6] AV. Marenich, SV. Jerome, CJ. Cramer, and DG. Truhlar, “Charge Model 5: An Extension of Hirshfeld Population Analysis for the Accurate Description of Molecular Interactions in Gaseous and Condensed Phases,” *Journal of chemical theory and computation* 8, no. 2 (2012): 527-541.

[7] G. Bussi, D. Donadio, and M. Parrinello, “Canonical Sampling Through Velocity Rescaling,” *The Journal of chemical physics* 126, no. 1 (2007).

[8] H. Yang, J. Yan, S. Gao, et al., “Asymmetric Functional Gel Polymer Electrolyte Enables Superior Interfacial Compatibility for Wide Temperature Lithium Metal Batteries,” *Energy & Environmental Science* (2025).

[9] L. Yu, S. Guo, Y. Lu, et al., “Highly Tough, Li‐Metal Compatible Organic–Inorganic Double‐Network Solvate Ionogel,” *Advanced Energy Materials* 9, no. 22 (2019): 1900257.

[10] J. Cui, Y. Liu, Y. Du, et al., “Self‐Regulating the Local Conjugation of Tertiary Aniline toward Highly Stable Polymer Li Metal Batteries,” *Advanced Materials* 37, no. 19 (2025): 2500876.

[11] G. Tang, SP. Shen, HJ. Li, et al., “Flame-Retardant Gel Electrolyte toward High-Safety Lithium Metal Batteries with High-Mass-Loading Cathodes,” *The Journal of Physical Chemistry C* 127, no. 20 (2023): 9463-9470.

[12] Y. He, X. Shan, Y. Li, et al., “In-Situ Formation of Quasi-Solid Polymer Electrolyte for Wide-Temperature Applicable Li-Metal Batteries,” *Energy Storage Materials* 68 (2024): 103281.

[13] J. Yu, J. Liu, X. Lin, et al., “A Solid-Like Dual-Salt Polymer Electrolyte for Li-Metal Batteries Capable of Stable Operation over an Extended Temperature Range,” *Energy Storage Materials* 37 (2021): 609-618.
